# Upregulation of iNOS Protects Cyclic Mechanical Stretch-Induced Cell Death in Rat Aorta Smooth Muscle Cells

**DOI:** 10.3390/ijms21228660

**Published:** 2020-11-17

**Authors:** Jing Zhao, Kiichi Nakahira, Akihiko Kimura, Yoji Kyotani, Masanori Yoshizumi

**Affiliations:** 1 Department of Pharmacology, Nara Medical University School of Medicine, 840 Shijo-Cho, Kashihara 634-8521, Japan; kin2019@naramed-u.ac.jp (K.N.); cd147@naramed-u.ac.jp (Y.K.); yoshizu@naramed-u.ac.jp (M.Y.); 2 Department of Forensic Medicine, Wakayama Medical University, 811-1 Kimiidera, Wakayama 641-8509, Japan; legkim@wakayama-med.ac.jp

**Keywords:** hypertension, NOS, stretch, vascular smooth muscle cell, cell death

## Abstract

Aortic dissection and aneurysm are associated with abnormal hemodynamic loads originating from hypertension. Our previous study demonstrated that cyclic mechanical stretch (CMS, mimicked hypertension) caused the death of rat aortic smooth muscle cells (RASMCs) in a mitogen activated-protein kinases (MAPKs)-dependent manner. The current study investigated the effects of inducible nitric oxide synthase (iNOS) on CMS-induced RASMC death. cDNA microarrays for CMS-treated RASMCs showed that iNOS expression levels were increased in response to CMS. Real-time polymerase chain reaction (PCR) analysis demonstrated that this increase was p38 MAPK (p38)-dependent. NO production was also increased. This increase could be inhibited by p38 and iNOS inhibitors. Thus, CMS-induced iNOS synthesized NO. CMS-induced cell death in RASMCs was increased by the iNOS inhibitor but abrogated by the long-acting NO donor DETA-NONOate. Increased iNOS expression was confirmed in the abdominal aortic constriction mouse model. Signal transducers and activators of transcription 1 (STAT1) was activated in stretched RASMCs, and iNOS expression and NO production were inhibited by the STAT1 inhibitor nifuroxazide. Our findings suggest that RASMCs were protected by iNOS from CMS-stimulated cell death through the STAT1 and p38 signal pathways independently.

## 1. Introduction

Both acute aortic dissection and ruptured aortic aneurysm are recognized as leading causes of death in cardiovascular disease [1,2]. Aortic dissection and aneurysm are characterized by progressive enlargement and acute dissection or rupture of the aorta and associated with abnormal hemodynamic loads originating from hypertension and weakness of the vascular wall [3,4]. Because blood flow is pulsatile, the hemodynamic load that occurs during hypertension exposes vascular smooth muscle cells (VSMCs) in the medial layers to cyclic mechanical stretch (CMS), which initiates VSMC proliferation, cell death, phenotypic switching, and migration that leads to vascular remodeling [5].

Many pathophysiological studies have shown that the amount of VSMCs substantially decreases after the onset of aortic dissection, indicative of the occurrence of VSMC death before and during an acute rise in blood pressure and subsequent aortic disease [6]. Significant effort has been made to clarify the possible causes of VSMC death induced by hypertension-related mechanical stretch [7,8,9,10]. Reactive oxygen species [11], nitric oxide (NO) [12], angiotensin II [13], and endothelin [14] are involved in vascular remodeling induced by hypertension. Alterations in any of these factors result in VSMC death, an adaptation of the vessel wall characterized by modified morphology and function. The activation of many kinases, such as MAPK, protein kinase C, Rho family GTPases, and phosphatidylinositol-3-kinase/Akt, are involved in the cellular response to mechanical stress [15,16,17,18,19]. However, the cellular mechanosensitive mechanisms are not fully understood, and it is still not clear which mechanobiological processes lead to VSMC death.

In our previous study, we reported that CMS mimicking a sudden rise in blood pressure in rat aortic smooth muscle cells (RASMCs) caused JNK- and p38-dependent cell death. Azelnidipine (a calcium channel blocker) and olmesartan (angiotensin II receptor antagonist) decreased the phosphorylation of JNK and p38 and RASMC death induced by CMS [20,21], independent of their blood-pressure-lowering effects. In addition, cDNA microarrays and real-time quantitative reverse transcription PCR (real-time RT-PCR) showed that the expression of inducible nitric oxide synthase (*iNOS*, *NOS2*), *chemokines*, and *NR4A1* dramatically changed in response to CMS [22].

iNOS can be a harmful enzyme in pathological conditions and is thought to be a major contributor to diseases of the cardiovascular system, such as atherosclerosis and hypertension [23,24]. However, some researchers have proposed that iNOS may protect VSMCs by inhibiting their proliferation and migration and stimulating endothelial cell growth [25]. Studies have shown that iNOS has a cardioprotective effect during ischemic preconditioning in mice [26,27], suggesting that iNOS has a controversial effect on vascular remodeling.

In the current study, we examined how iNOS affected CMS-induced RASMC death and discussed a possible mechanism underlying the iNOS effects.

## 2. Results

### 2.1. Transcriptome Analysis Revealed a Potential Association between MAPKs, Apoptosis, and Aneurysm

**Figure 1:**
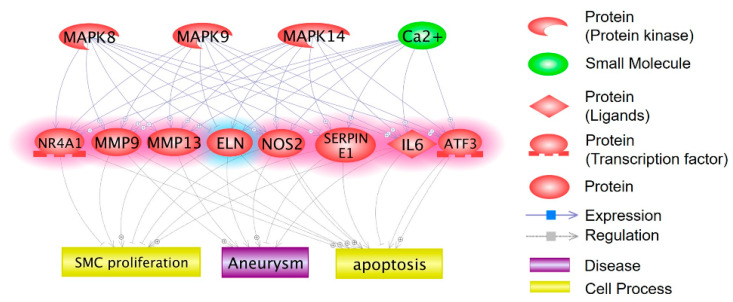
Molecular networks associated with mitogen activated-protein kinases (MAPKs) (JNK and p38), apoptosis, and aneurysm. The 91 differentially expressed genes (DEGs) dysregulated in rat aortic smooth muscle cells (RASMCs) treated with cyclic mechanical stretch (CMS) were subjected to bioinformatics analyses to identify genes related to both the JNK and p38, apoptosis, and aneurysm. The regulation between the MAPKs, dysregulated genes, apoptosis, and aneurysm is shown in the network.

We previously demonstrated that JNK (MAPK8 and MAPK9) and p38 (MAPK14) were involved in the death of RASMCs induced by CMS [20]. Using cDNA microarrays and bioinformatics analysis, we identified 91 differentially expressed genes (DEGs) in RASMCs following CMS. Of these DEGs, 29 DEGs were potentially regulated by JNK or p38 and related to cell death [22]. In the current study, we used Pathway Studio [28] on the 91 DEGs to identify genes related to RASMC death regulated by MAPKs and diseases. This analysis was also used to identify potential expression regulators, cell processes, and diseases related to the 91 genes. As shown in Table S1, 100 cell processes were identified, including cell death (*p* = 3.54 × 10^−11^), apoptosis (*p* = 1.34 × 10^−13^), proliferation (*p* = 7.26 × 10^−16^), and calcium ion homeostasis (*p* = 2.73 × 10^−10^). Furthermore, diseases were identified, including aneurysm (*p* = 1.03 × 10^−11^), as shown in Table S2. Moreover, the results revealed that eight of the 91 DEGs were potentially regulated by MAPK8, MAPK9, or MAPK14 and related to VSMC death and aneurysm. The molecular networks are shown in Figure 1.

### 2.2. Cyclic Mechanical Stretch Enhances iNOS Expression

**Figure 2:**
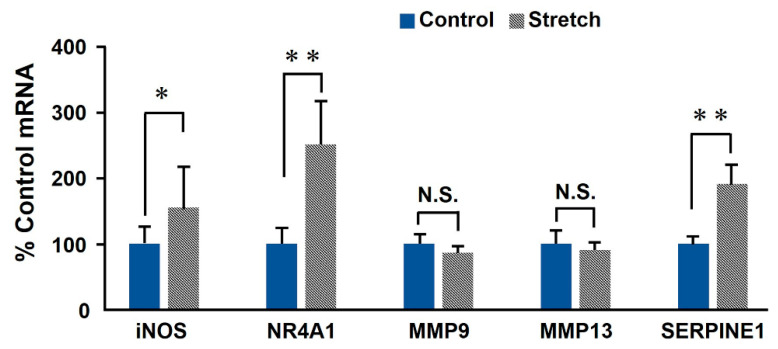
Expression of candidate genes in RASMCs subjected to CMS. RASMCs were subjected to CMS for 4 h, and the expression levels of *iNOS, NR4A1, MMM9, MMP13,* and *SERPINE1* were evaluated by real-time RT-PCR. The quantity of the transcripts is expressed as a percentage of the control, normalized to *GAPDH*. Data are presented as the means ± SD (*n* = 6); * *p* < 0.05 and ** *p* < 0.01 versus control; N.S. indicates no statistically significant difference.

We used real-time RT-PCR to evaluate the expression of some genes potentially involved in RASMC death and aneurysm to confirm the results obtained from the cDNA microarray and transcriptome analysis. Transcripts of *iNOS, NR4A1, SERPINE1, MMP9,* and *MMP13* were differentially expressed in cyclically-stretched RASMCs (Figure 2). *iNOS, NR4A1, and SERPINE1* expression levels were significantly increased in RASMCs subjected to CMS, whereas those of *MMP9* and *MMP13* did not significantly change relative to the control and CMS-treated RASMCs. In the following investigation, we focused on the role of *iNOS* in CMS-induced cell death, irrespective of the effects of *NR4A1* and *SERPINE1*.

### 2.3. CMS Changes iNOS Expression and NO Production in a p38-Dependent Manner

**Figure 3:**
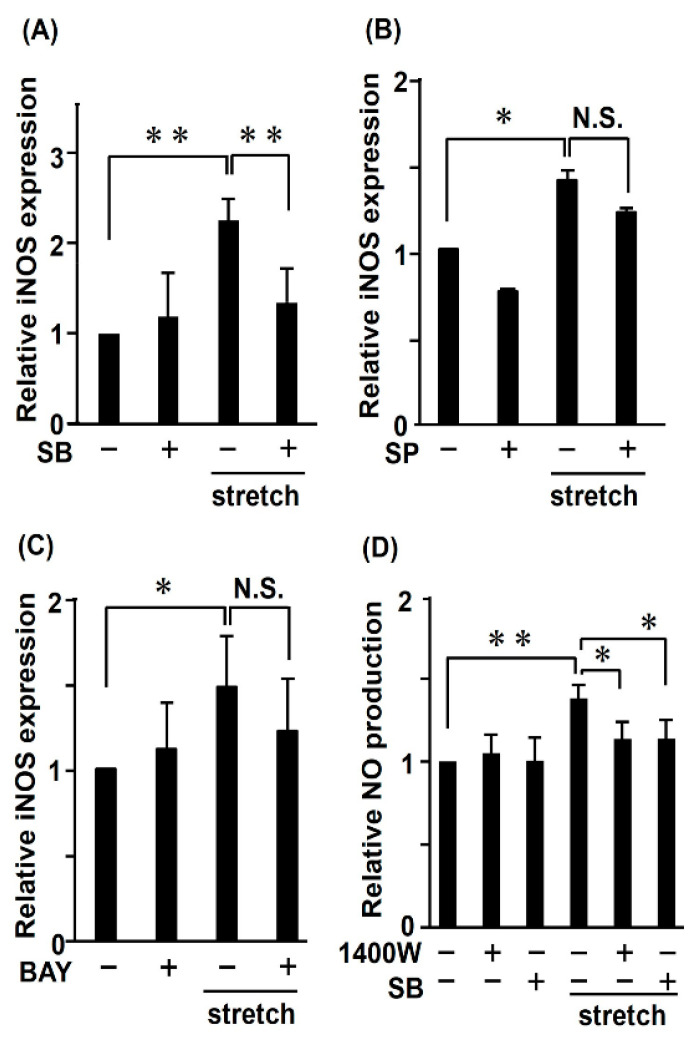
Induction of inducible nitric oxide synthase (iNOS) and NO in RASMCs subjected to CMS. RASMCs were incubated with SB203580 (SB, 20 μM, 20 min) (**A**), SP600125 (SP, 20 μM, 20 min) (**B**), BAY11-7082 (BAY, 2.5 µM, 20 min) (**C**), or 1400 W (50 nM, 1 h) (**D**) followed by CMS for 4 h. Cells were harvested and analyzed by real-time RT-PCR with specific primers for *iNOS* (**A**–**C**) and the medium was analyzed for NO production by ELISA (**D**). Data are means ± SD (*n* = 9); * *p* < 0.05 and ** *p* < 0.01 versus CMS control; N.S. indicates no significant difference.

The viability of RASMCs subjected to CMS decreased in a JNK- and p38-dependent manner [21]. Therefore, we determined the effects of CMS on *iNOS* expression and MAPKs by real-time RT-PCR. We first evaluated the *iNOS* mRNA and using JNK and p38 inhibitors (SP600125 and SB203580, respectively). Notably, the p38 inhibitor SB203580 caused an approximately 40% decrease in *iNOS* transcript levels compared to cells subjected to CMS in the absence of p38 inhibitor, indicating that *iNOS* expression is transcriptionally regulated by p38 (Figure 3A). In contrast, SP600125 did not affect *iNOS* expression (Figure 3B). *iNOS* is a known nuclear factor-kappaB (NF-κB)-responsive gene regulated by various kinases that cause the nuclear translocation of NF-κB [29]. To investigate whether CMS-induced *iNOS* expression is regulated by the NF-κB pathway, we pretreated RASMCs with an IKK (NF-κB essential modulator) inhibitor (BAY11-7082) before CMS. We found that BAY11-7082 did not affect *iNOS* expression (Figure 3C). The results suggest that the induction of *iNOS* gene expression by CMS might be associated with and controlled by p38.

Because iNOS plays its role in vascular diseases by catalyzing NO production [30], we measured NO levels by determining the amount of nitrite released into the cultured medium following treatment. NO production was inhibited to the same extent by both the p38 and iNOS inhibitors (SB203580 and 1400 W, respectively) in CMS-treated RASMCs, indicating that CMS-induced *iNOS* expression and NO production were both p38-dependent (Figure 3A,D).

### 2.4. CMS-Induced RASMC Death Was Increased by iNOS Inhibitor and Decreased by NO Donor

**Figure 4:**
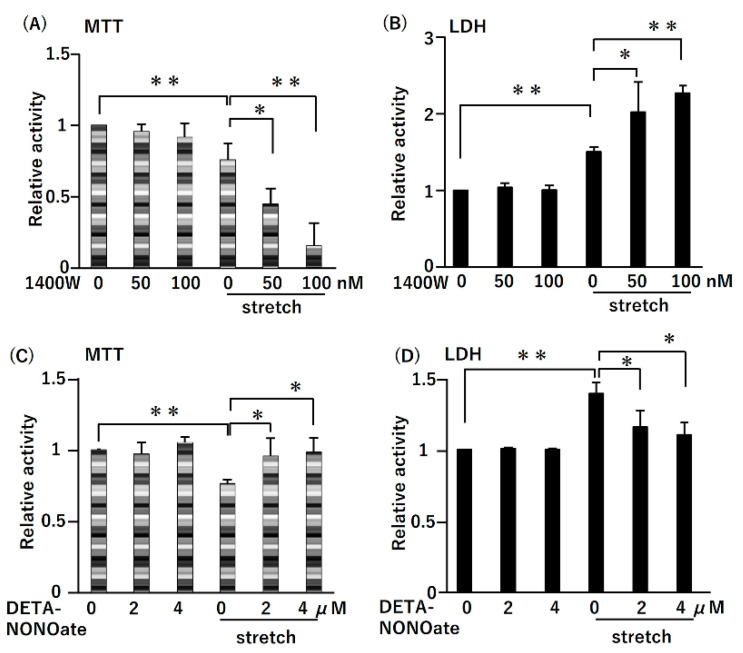
Effects of iNOS inhibitor and NO donor on CMS-induced cell death in RASMCs. The RASMCs were pretreated with iNOS inhibitor (1400 W, 1 h) or NO donor (DETA-NONOate, 20 min) at the indicated concentrations and then incubated under normal conditions or subjected to CMS for 4 h. After CMS, the cells were incubated for 24 h, and then cell viability and death were evaluated by the MTT (**A**,**C**) and LDH (**B**,**D**) assays, respectively. Colorimetric analysis of each value was normalized by arbitrarily setting the absorbance value of the control to 1. Data are presented as the mean ± SD (*n* = 9); * *p* < 0.05 and ** *p* < 0.01 versus the CMS control.

We examined the effects of iNOS and NO on CMS-induced cell death in RASMCs (Figure 4). The pretreatment of RASMCs with iNOS inhibitor (50 nM) decreased viability by 30% compared to RASMCs treated with CMS alone (Figure 4A). These data were confirmed by the LDH assay (Figure 4B). In contrast, the long-acting NO donor DETA-NONOate (2 µM) improved cell viability by 25% (Figure 4C) and attenuated the cell death rate of the RASMCs (Figure 4D). Because DETA-NONOate has a half-life of about 20 h at 37 °C, a steady release of low amounts of NO by DETA-NONOate protects against CMS-induced RASMC death. These results indicate that *iNOS* expression and NO production could inhibit CMS-induced RASMCs death in a concentration-dependent manner.

### 2.5. Hypertension Induces iNOS Expression in Vascular Smooth Muscle Cells

**Figure 5:**
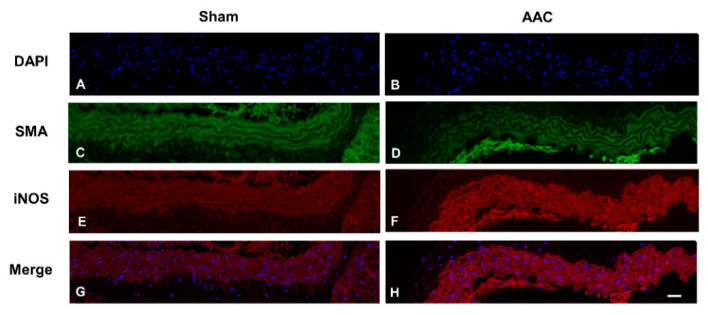
Abdominal aortic constriction (AAC)-induced iNOS expression in the aorta of mice. Immunofluorescence images of iNOS (red) and smooth muscle actin (SMA, green) in the aortas of sham and AAC mice 6 h post-operation. Tissue sections were co-stained with DAPI (**A**,**B**, blue), anti-SMA (**C**,**D**) and anti-iNOS (**E**,**F**) antibodies. The Merge is the overlapping of iNOS and DAPI (**G**,**H**). The uncropped pictures of immunohistochemical analysis are shown in Figure S1. Representative images from four individual animals in each group are shown. Original magnification, 400× (scale: 20 µm).

Pulsatile blood pressure creates hemodynamic stimuli (e.g., CMS) that act on the constituents of blood vessel walls. To determine whether hypertension could induce iNOS expression in vivo, we evaluated iNOS expression in VSMCs using an abdominal aortic constriction (AAC) mouse model, which induces sustained elevation of mean arterial pressure without the use of a vasopressor. We observed that iNOS protein was increased in arteries subjected to AAC (Figure 5E,F) compared to sham-operated arteries (Figure 5C,D). These results are consistent with those for RASMCs subjected to CMS, suggesting that iNOS expression in VSMCs is likely induced in the early stages of hypertension.

### 2.6. STAT1 Mediates CMS-Induced iNOS Expression and NO Production

**Figure 6:**
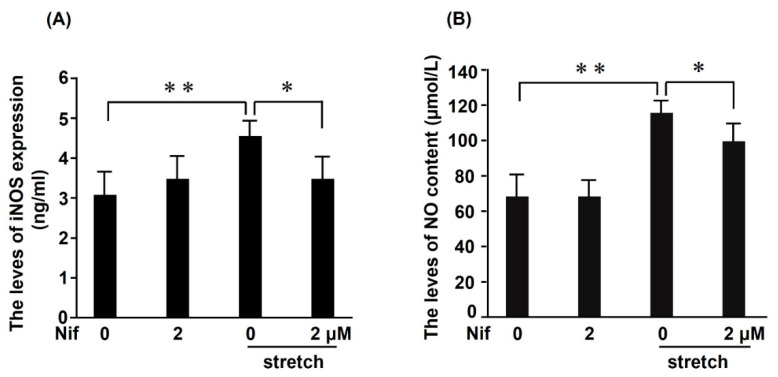
Effects of signal transducers and activators of transcription 1 (STAT1) inhibitor on iNOS protein expression and NO production induced by CMS in RASMCs. RASMCs were incubated with a STAT1 inhibitor (nifuroxazide; Nif) at the indicated concentrations for 1 h and then incubated under normal conditions or subjected to CMS for 4 h. After CMS, the cells and medium were harvested, and iNOS protein expression (ng/mL) and NO production (µmol/L) were evaluated by ELISA (**A**) and the NO colorimetric assay (**B**), respectively. Data are presented as the mean ± SD (*n* = 9); * *p* < 0.05 and ** *p* < 0.01 versus the CMS control.

Our previous studies revealed that CMS stimulates phosphorylation of STAT1 in RASMCs, and STAT1 is involved in CMS-induced cell death signaling gene transcription [22]. To clarify whether STAT1 is involved in regulating iNOS expression induced by CMS, we pretreated RASMCs with the STAT1 selective inhibitor nifuroxazide. This pretreatment significantly decreased iNOS protein expression (Figure 6A) and NO production (Figure 6B) in RASMCs subjected to CMS, demonstrating that STAT1 activation might be indispensable for the induction of iNOS.

### 2.7. CMS-Induced iNOS-NO Signaling Involves Phosphorylation of STAT1 and p38

**Figure 7:**
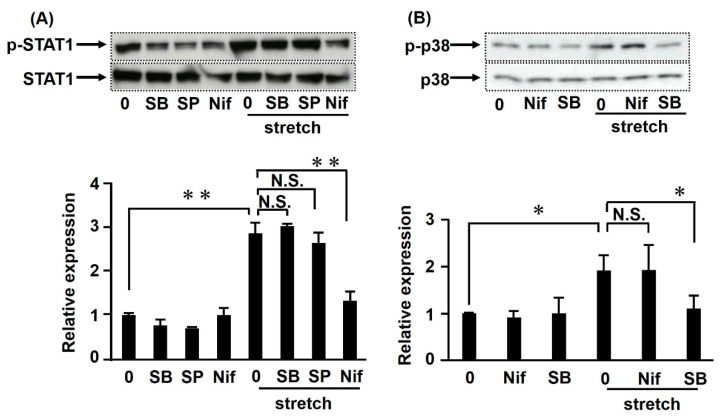
Effects of p38 and STAT1 inhibitors on STAT1 and p38 phosphorylation induced by CMS in RASMCs. RASMCs were pretreated with SB203580 (SB, 20 µM, 20 min), SP600125 (SP, 20 μM, 20 min), or nifuroxazide (Nif, 2 µM, 1 h) and then subjected to CMS for 4 h. Cell lysates were analyzed by immunoblotting using anti-phosphorylated and total STAT1 antibodies (**A**) and anti-phosphorylated and total p38 antibodies (**B**). Phosphorylated STAT1 or p38 levels are expressed as a percentage of the control, normalized to total STAT1 or p38, respectively. The uncropped pictures of immunoblotting are shown in Figure S2. Data are presented as the mean ± SD (*n* = 3); * *p* < 0.05 and ** *p* < 0.01 versus the CMS control; N.S. indicates no significant difference.

The above results demonstrated that p38 and STAT1 mediated iNOS-NO signaling induced by CMS. To clarify the mechanism of how STAT1 and p38 are involved in CMS-regulated iNOS-NO signaling in RASMCs, we performed Western blot analysis to evaluate their potential association. Immunoblotting confirmed that STAT1 was phosphorylated in RASMCs subjected to CMS (Figure 7A). Furthermore, we evaluated the influence of STAT1 and p38 inhibitors on the phosphorylation of p38 and STAT1 in stretched RASMCs. Interestingly, the p38 inhibitor SB203580 did not affect the phosphorylation of STAT1; and the STAT1 inhibitor nifuroxazide did not alter the phosphorylation of p38 (Figure 7B). These data suggest that STAT1 and p38 might independently regulate iNOS-NO signaling.

## 3. Discussion

The nitric oxide synthases are a family of enzymes that catalyze the production of NO, including eNOS, nNOS, and iNOS. NO generated in RASMCs contributes to vasculature homeostasis. Because NO has quite a short duration of action (i.e., a half-life of only a few seconds), most studies on its physiological functions focus on the regulation of NOS activity. Of the three enzymes, iNOS genes are not easily expressed under physiological conditions. iNOS expression can be induced in macrophages [31], endothelial cells [32], vascular smooth muscle cells [33], and cardiac myocytes [34] after stimulation with lipopolysaccharide or cytokines. These studies suggest that increased iNOS expression and activity contribute to the pathogenesis of hypertension and its complications. In the current study, we found that CMS stimulated iNOS expression and NO production in RASMCs, suggesting that iNOS is a potential pharmacological target for the treatment of hypertension.

As a unique messenger in cell signaling, NO plays a role not only in protecting against but also in initiating human cardiovascular disease. NO is a principal and potent mediator of vasodilation, and low amounts of NO produced in cells facilitate cardiovascular homeostasis. However, high NO levels may be detrimental to the cardiovascular system and contribute to hypertension [35]. We found that CMS treatment of RASMCs caused an approximately 50% increase in *iNOS* expression and a corresponding increase in NO (~40%). Based on these results, low NO concentrations could be beneficial to cell function. NOS has three isoforms, and the eNOS isoform is expressed in smooth muscle cells of the arteries [36]. Therefore, we examined the eNOS expression levels. However, the expression of this isoform did not change in RASMCs following CMS (Figure S3), suggesting that iNOS was responsible for the NO production observed in this study.

To explore whether CMS-induced iNOS expression and NO production were directly related to CMS-induced RASMC death, we pretreated the RASMCs with the iNOS inhibitor 1400 W. We found that RASMC death increased significantly with increasing concentrations of 1400 W. Conversely, RASMC death decreased with increasing concentrations of NO donor DETA-NONOate. These results demonstrated that NO generated by iNOS was dependent to a significant extent on the expression levels of the enzyme. Thus, the stretching load could induce iNOS expression and subsequent NO production to protect RASMCs from CMS-induced cell death.

NO is an autocrine and paracrine signaling molecule that can penetrate cell membranes and affect adjacent cells. Downstream targets of NO include guanylyl cyclase (GCs) and NF-κB [37]. The latter is a transcription factor involved in *iNOS* gene expression [38,39]. In addition to NF-κB, activation of the transcription factor STAT1 and subsequent activation of the *iNOS* promoter are essential steps for iNOS induction in most human cells [40,41]. Moreover, the bioinformatics analysis performed on CMS-treated and untreated RASMCs identified STAT1 as a master transcription factor that potentially regulates 11 DEGs, including *iNOS*. However, whether, how, and to what extent STAT1 phosphorylation affects iNOS-NO signaling induced by CMS remains unclear. To determine if NF-κB and STAT1 are involved in regulating iNOS expression induced by CMS, we pretreated RASMCs with NF-κB and STAT1 inhibitors and examined CMS-induced iNOS expression and NO production. The STAT1 inhibitor significantly reduced the induction of iNOS expression and NO production in RASMCs subjected to CMS, whereas pretreatment with the NF-κB inhibitor had little or no effect on the iNOS and NO. Therefore, it is conceivable that STAT1 phosphorylation, but not the NF-κB pathway, could play a positive role in iNOS-NO signaling induced by CMS.

Studies by our laboratory and others have shown that mechanical forces can increase p38 phosphorylation [20,21,22]. In the present study, inhibitors of both p38 and STAT1 could reduce the induction of iNOS expression and NO production in RASMCs caused by CMS, suggesting that both factors were involved in CMS-mediated iNOS-NO signaling. However, the p38 inhibitor had little or no effect on CMS-induced STAT1 phosphorylation and vice versa. This may be explained by the fact that STAT1 and p38 might independently regulate iNOS-NO signaling.

Based on our findings, we suggest that the induction of iNOS could provide a protective effect against the death of VSMCs in the early phase of a sudden rise in blood pressure, which might inhibit vascular remodeling. However, high NO levels associated with iNOS activity may have detrimental consequences on the cardiovascular system as a mediator of the adaptive response, contributing to hypertension [42,43,44]. These findings suggest a novel approach for the prevention and treatment of aortic dissection and aneurysm caused by acute hypertension at an early stage.

## 4. Materials and Methods

### 4.1. Study Approval and Material Preparation

All methods were performed in accordance with relevant guidelines and regulations. All experimental protocols were approved by the institutional committee. Materials were purchased from Wako (Kyoto, Japan) or Nacalai Tesque (Kyoto, Japan) unless stated otherwise. Collagen I was purchased from Nippon Meat Packers, Inc. (Osaka, Japan). The antibodies used for Western blotting were as follows: Anti-phospho-SAPK/JNK (Thr183/Tyr185) antibody and anti-phospho-p38 MAP kinase (Thr180/Tyr182) antibody were purchased from Cell Signaling Technology (Tokyo, Japan); the anti-iNOS antibody and Alexa-conjugated donkey anti-rabbit IgG polyclonal antibody were purchased from Abcam Technology (Tokyo, Japan); the smooth muscle actin (SMA) antibody was purchased from Dako (Carpinteria, CA, USA); FITC-conjugated donkey anti-mouse IgG polyclonal antibodies and the p-STAT1 (A-2) antibody were purchased from Santa Cruz Biotechnology Inc. (Dallas, Texas, USA). The ECL plus system was purchased from GE Healthcare (Tokyo, Japan). Nifuroxazide was purchased from Selleckchem. Inc. (Tokyo, Japan). The 1400 W was purchased from Tocris Bioscience (Minneapolis, MN, USA). DETA-NONOate was purchased from Cayman Chemical (Ann Arbor, MI, USA). Nuclear Decloaker buffer was purchased from Biocare Medical (Concord, CA, USA). All chemical compounds were dissolved in dimethyl sulfoxide (DMSO) at a final concentration of less than 1% unless otherwise specified. The Nitric Oxide Synthase 2 (NOS2) ELISA kit was purchased from Cloud-Clone Corp. (Wuhan, China). The nitric oxide colorimetric assay kit was purchased from BioVision Inc. (Milpitas, CA, USA).

### 4.2. Culture of RASMCs

This study was conducted following the Guide for the Care and Use of Laboratory Animals as accepted and endorsed by the United States National Institutes of Health, with approval from the Ethics Committee of Nara Medical University (No. 11011). Isolation of RASMCs from the thoracic aortas of male Sprague–Dawley rats (8 weeks of age) was performed as previously described [20]. The RASMCs were maintained in Dulbecco’s Modified Eagle’s Medium (DMEM) containing 10% fetal bovine serum (FBS, HyClone, Logan, UT, USA), 100 U/mL penicillin, and 100 mg/mL streptomycin at 37 °C with 5% CO_2_ in a humidified incubator and used for experiments between passages 3 and 6.

### 4.3. Effects of In Vitro CMS on Cultured RASMCs

The primary cells were cultured in collagen I-coated silicon chambers (5 × 10^3^ cells/mm^2^) (STREX Inc., Osaka, Japan). When confluency reached 70–80%, the cells were cultured for an additional 24 h in serum-free DMEM. After the incubation, the RASMCs were randomly assigned to the test groups. CMS (60 cycles/min, 15% elongation) was performed for 4 h using the computer-controlled mechanical strain unit (STREX Inc., Osaka, Japan). After cyclic stretch, the medium was replaced with DMEM containing 0.1% FBS.

To detect cell viability, RASMCs were pretreated with or without chemical inhibitors and then subjected to CMS for 4 h. The specific pretreatment details described in the corresponding results section. After incubating for 24 h post-treatment, the cells were subjected to the MTT assay, and the culture medium assayed for the release of LDH and NO production. Cell lysates were also assayed for iNOS protein expression.

### 4.4. RNA Isolation and Gene Expression Analysis

Isolation of total RNA from RASMCs was performed using the RNeasy Plus Mini Kit, as described previously [22]. RNA concentration and integrity were assessed using the Agilent 2100 Bioanalyzer (Agilent Technologies, Palo Alto, CA, USA). The Low Input Quick Amp Labeling Kit (Agilent Technologies, Santa Clara, CA, USA) was used to amplify and label the RNA samples for hybridization, according to the manufacturer’s instructions. Two control and two CMS-subjected RASMC samples were analyzed with Rat 4 × 44 K Ver.3.0 (Agilent Technologies, Santa Clara, CA, USA).

### 4.5. Real-Time Quantitative Reverse Transcription PCR (Real-Time RT-PCR)

Total RNA was reverse- transcribed using PrimeScript™ Reverse Transcriptase (Takara, Japan), according to the manufacturer’s instructions. The cDNA was used as a template for SYBR Green quantitative real-time PCR using the THUNDERBIRD^®^ SYBR qPCR Mix (Toyobo, Japan). Reactions were performed using the StepOnePlus Real-Time PCR System (Applied Biosystems, Carlsbad, CA, USA). The relative amount of target mRNA was calculated after normalization to GAPDH. The sequences of primers used for the real-time RT-PCR are presented in Table 1. Data are presented as the mean ± standard deviation (SD).

### 4.6. MTT and LDH Assays

The MTT assay method was previously described [20]. Briefly, MTT solution (50 μg, Sigma-Aldrich, St. Louis, MO, USA) was added to each well. Following incubation at 37 °C for 1 h, the cells were solubilized with 50% acidified *N*,*N*-dimethylformamide (DMF, pH 4.7). The optical density (540 nm) was measured using a microplate reader (Thermo Scientific, Waltham, MA, USA). The LDH assay was carried out according to the manufacturer’s instructions using the Cytotoxicity Detection kit (Roche Diagnostics, Penzberg, Germany).

### 4.7. Western Blot Analysis

Immunoblotting was performed as described previously [22]. Briefly, total protein was separated by sodium dodecyl sulfate-polyacrylamide gel electrophoresis (SDS-PAGE) and transferred onto polyvinylidene difluoride (PVDF) membranes. The membranes were blocked with 5% skim milk in Tris-buffered saline with 0.01% Tween 20 (TBS-T) and then incubated with primary antibody. After washing with TBS-T, the membranes were incubated with a secondary antibody in TBS-T containing 5% skim milk. After washing, the blots were developed with ECL plus Western blotting substrate (Buckinghamshire, UK). Band intensities were quantified using NIH ImageJ software (version 1.52p, https://imagej.nih.gov/ij/; provided in the public domain by the National Institutes of Health, Bethesda, MD, USA).

### 4.8. Measurement of iNOS Protein Expression and NO Production

RASMCs were pretreated with or without various inhibitors and then subjected to CMS for 4 h. The specific pretreatment details are described in the corresponding results section. Cell lysates were collected and assayed for iNOS protein expression by ELISA, according to the manufacturer’s instructions. The BioVision Nitric Oxide Colorimetric Assay Kit was used to determine NO production, according to the manufacturer’s instructions. NO is rapidly oxidized to nitrite and nitrate. Thus, these two metabolites in medium are used to quantitate NO production.

### 4.9. Abdominal Aortic Constriction (AAC) Protocol

All experimental procedures were approved by the Animal Research Committee of Wakayama Medical University as described previously [22]. AAC was performed to induce hypertension. The mice were anesthetized with 1.5% isoflurane (Mylan, Japan), and a longitudinal skin incision was made on the left lateral side of the abdomen. The abdominal aorta was constricted with a 7–0 silk suture tied firmly two times against a 28-gauge blunted needle. After ligation, the needle was removed, the skin was closed, and the mice were allowed to recover. Sham-operated mice underwent a similar surgical procedure without the constriction of the aorta. Six hours after AAC, thoracic aortas were collected from AAC and sham-operated mice and subjected to immunohistochemical analyses.

### 4.10. Immunohistochemical Analysis

Double-color immunofluorescence analysis was performed to determine iNOS expression levels in mouse aorta tissue, as described previously [22]. The aortas were fixed in 4% formaldehyde buffered with PBS (pH 7.2) to prepare paraffin-embedded sections (4 μm thick). Tissue sections were deparaffinized, retrieved with heat in Nuclear Decloaker buffer (EDTA/Tris buffer, pH 9.5). The slides were incubated with PBS containing 1% normal horse serum and 1% BSA to reduce nonspecific reactions. Thereafter, the sections were incubated with rabbit anti-iNOS polyclonal antibodies and mouse anti-human SMA monoclonal antibodies both at 1:100 dilution at 4 °C overnight. After washing, the sections were incubated with Alexa-conjugated donkey anti-rabbit IgG polyclonal antibodies (1:500) and FITC-conjugated donkey anti-mouse IgG polyclonal antibodies (1:500) at room temperature for 1 h. The slides were then observed using a fluorescence microscope (KEYENCE BZ-X700, Osaka, Japan).

### 4.11. Statistical Analysis

The Student’s *t*-test was used to compare the differences between two groups. One-way ANOVA, followed by Tukey’s HSD test, was used to compare the differences between multiple groups. Differences were considered significant with a *p* < 0.05.

## Figures and Tables

**Table 1:** Primers used for the real-time RT-PCR.

Gene Name	Reverse Primer Sequence (5′-3′)	Forward Primer Sequence (5′-3′)
*iNOS*	cagcatccacgccaagaa	caggtgttccccaggtaggtag
*NR4A1*	ccttgggatggttcatttgg	gtgggaggactgaaggagaaga
*MMP9*	cggatggttatcgctggtg	caggaagacgaaggggaaga
*MMP13*	accctggagccctgatgtt	ctctggtgttttggggtgct
*SERPINE1*	gcacaagcactacaaaaggtcaag	tgccgaaccacaaagagaaa
*eNOS*	ttcacaggcttcgccatc	aaaggcacaggcatcacca
*GAPDH*	cagcaaggatactgagagcaagag	gttatggggtctgggatgga

## Supplementary Materials

The following are available online at https://www.mdpi.com/1422-0067/21/22/8660/s1. Figure S1: Uncropped pictures of immunohistochemical analysis, Figure S2: Uncropped pictures of immunoblotting, Figure S3: Induction of eNOS in RASMCs subjected to CMS, Table S1: Cell processes significantly related to the 91 DEGs in RASMC treated with CMS, Table S2: Enrichment analysis of the DEGs focusing on “Diseases”.

## Abbreviations

JNK	Jun amino terminal kinase
NR4A1	nuclear receptor subfamily 4 group A member 1
MMP9	matrix metalloproteinase 9
MMP13	matrix metalloproteinase 13
SERPINE1	serpin family E member 1
eNOS	endothelia nitric oxide synthase
nNOS	nitric oxide synthase 1(neuronal)
GAPDH	glyceraldehyde-3-phosphate dehydrogenase
DAPI	4′,6-diamidino-2-phenylindole
MTT	3-(4,5-Dimethylthial-2-yl)-2,5-Diphenyltetrazalium Bromide
LDH	lactate dehydrogenase
ELISA	enzyme-linked immunosorbent assay
PBS	phosphate-buffered saline
