# Supplementary material for: Upregulation of iNOS Protects Cyclic Mechanical Stretch-Induced Cell Death in Rat Aorta Smooth Muscle Cells
Source: Int J Mol Sci. 2020 Nov 17;21(22):8660. doi: 10.3390/ijms21228660 (PMC7698365; doi:10.3390/ijms21228660)
Supplement: Supplementary file 1 [file ijms-21-08660-s001.pdf]

Upregulation of iNOS Protects Cyclic Mechanical Stretch-Induced Cell Death in Rat Aorta Smooth Muscle Cells

Jing Zhao[1,*], Kiichi Nakahira[1], Akihiko Kimura[2], Yoji Kyotani[1], Masanori Yoshizumi[1]

[1] Department of Pharmacology, Nara Medical University School of Medicine, 840 Shijo-Cho, Kashihara 634-8521, Japan; kin2019@naramed-u.ac.jp (K.N.); cd147@naramed-u.ac.jp (Y.K.); yoshizu@naramed-u.ac.jp (M.Y.)

[2] Department of Forensic Medicine, Wakayama Medical University, 811-1 Kimiidera, Wakayama 641-8509, Japan; legkim@wakayama-med.ac.jp

* Correspondence: jingzhao@naramed-u.ac.jp; Tel.: +81-744-22-3051; FAX: +81-744-29-0510

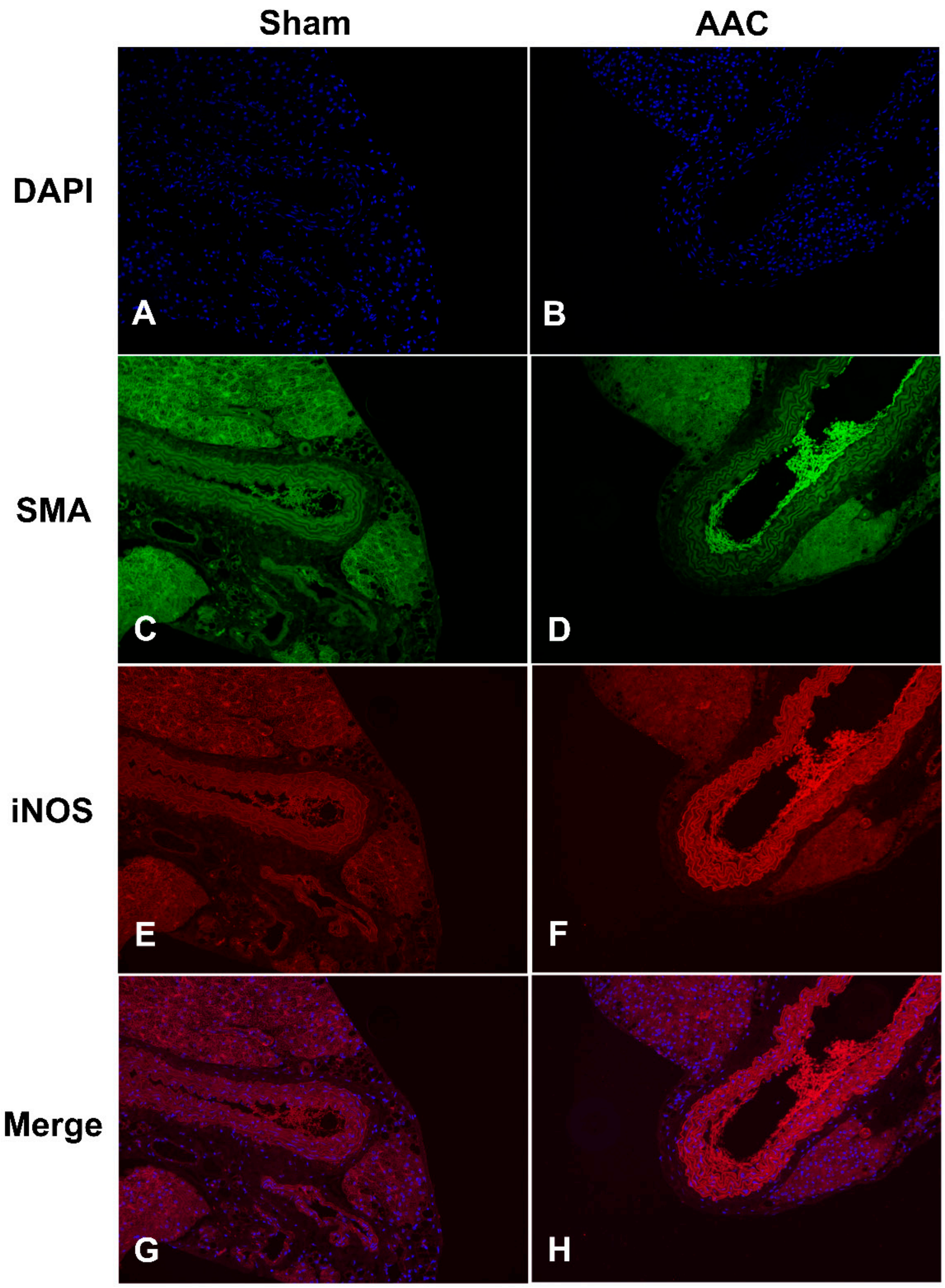

Supplemental Figure S1. Uncropped pictures of immunohistochemical analysis. Uncropped pictures of Figure 5 were shown.

(A)

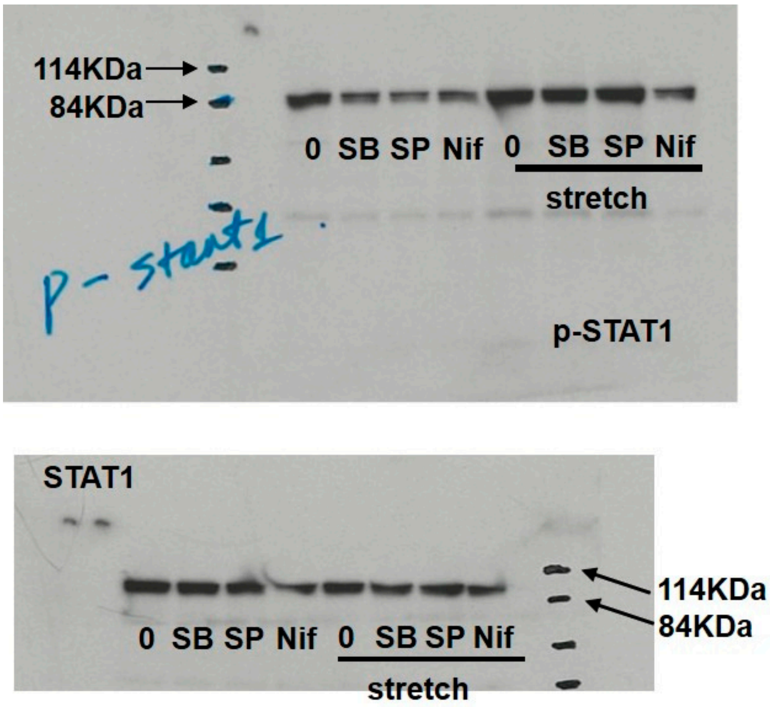

(B)

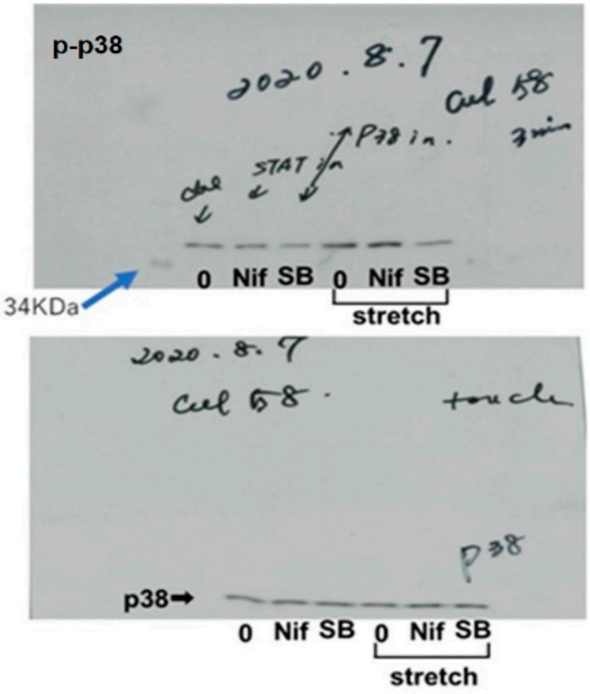

Supplemental Figure S2. Uncropped pictures of immunoblotting.

Uncropped pictures of Figure 7 were shown.

(A)

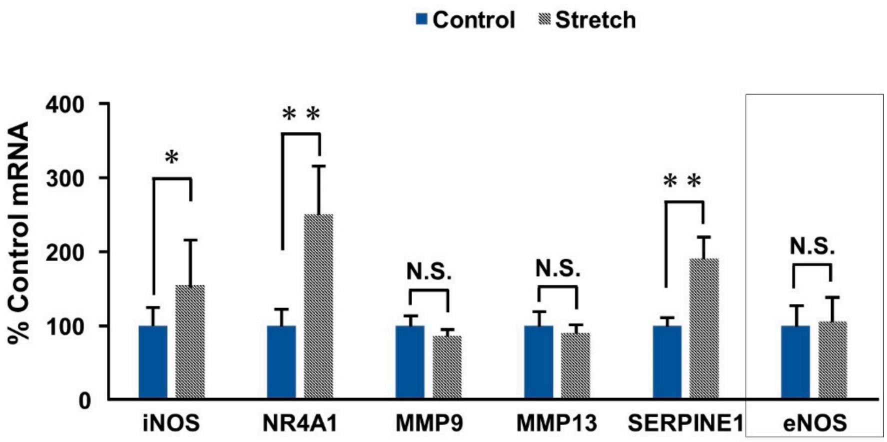

(B)

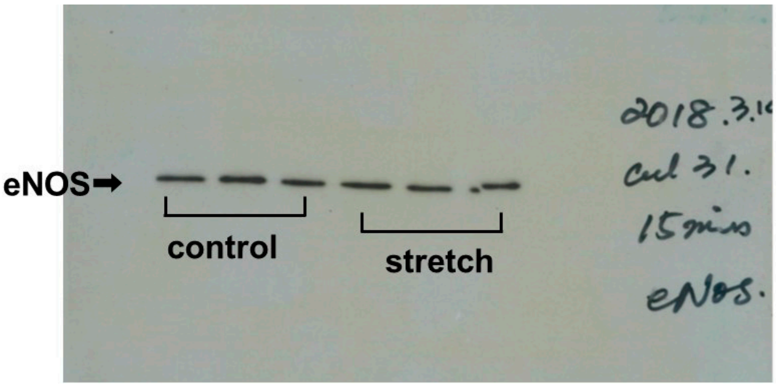

Supplemental Figure S3. Induction of eNOS in RASMCs subjected to CMS.

RASMCs were subjected to CMS for 4 h. Cells were harvested and analyzed by real-time RT-PCR with specific primers (A) and by western blot analysis (B) for *eNOS*. All values represent means ± SD (n = 6). N.S. indicates no significant difference.

Table S1-1. Cell processes significantly related to the 91 DEGs in RASMC treated with CMS

	cell process	p-value	Overlapping Entities
1	inflammatory response	1.77851E-17	NOS2;SPRY1;BDKRB1;SELP;MMP3;IL6;CXCL2;APOC2;CXCL5;STEAP4;ELN;CXCL1;CXCL3;ZC3H12A;CD180;SMPD3;C3;MMP13;TNFRSF9;PTGES;SNAI2;LCN2;NR4A1;PCP4;TLR2;TNFRSF12A;FCER1G;MMP9;CX3CL1;SLPI;NPPB;TSC22D3;SERPINE1;DDIT3;STAR;NFKBIZ;HP;ATF3;ABCG1;PLA2G2A;CCL7;TNIP3;NR4A3;PTX3;MT2A
2	cell growth	3.89315E-16	SPRY1;OGN;CXCL2;APOC2;CXCL5;STEAP4;TFRC;CXCL3;CD180;DUSP5;RGS2;BANP;SMPD3;C1QTNF3;FLRT3;BTG2;TNFRSF9;LAMC2;EHBP1;NR4A1;TLR2;TNFRSF12A;MMP9;CX3CL1;PLN;NPPB;SH3KBP1;NFKBIZ;ATF3;PRG4;CCL7;GJA5;RASD1;FOXK2;NR4A3;NOS2;BDKRB1;CYP7B1;CFLAR;MT1A;TFPI2;MMP3;IL6;FANCD2;RNASE1;HPGD;ELN;CXCL1;SH3BP4;TERC;SLC4A11;MMP13;EGLN3;PTGES;LCN2;FGF9;PDE1A;SLPI;TSC22D3;SERPINE1;CYP26B1;DDIT3;HP;PLA2G2A;Tmeff2;MT2A
3	**SMC proliferation**	**7.26413E-16**	**NOS2;BDKRB1;SELP;SLC16A3;TFPI2;MMP3;IL6;ELN;ZC3H12A;SMPD3;C1QTNF3;MMP13;TNFRSF9;PTGES;NR4A1;TLR2;TNFRSF12A;FGF9;MMP9;PDE1A;CX3CL1;NPPB;SERPINE1;CYP26B1;DDIT3;CCL7;NR4A3;PTX3**
4	immune response	1.56728E-15	NOS2;SPRY1;BDKRB1;CFLAR;SELP;IL6;CXCL2;FANCD2;CXCL5;STEAP4;HPGD;ELN;CXCL1;TFRC;CXCL3;ZC3H12A;CD180;C3;TNFRSF9;PTGES;LAMC2;LCN2;UAP1;NR4A1;TLR2;TNFRSF12A;FCER1G;MMP9;CX3CL1;SLPI;NPPB;TSC22D3;SERPINE1;RASL11B;STAR;SH3KBP1;NUP210;Fv1;NFKBIZ;HP;ATF3;ABCG1;PRG4;PLA2G2A;CCL7;PTX3
5	cell proliferation	2.3931E-14	SPRY1;SELP;LYPD1;G0S2;OGN;CXCL2;CXCL5;STEAP4;TFRC;CXCL3;H2AFY2;DUSP5;CD180;RGS2;SMPD3;C1QTNF3;FLRT3;BTG2;TGM1;TNFRSF9;GALNT14;LAMC2;UAP1;SNAI2;NR4A1;TLR2;TNFRSF12A;MMP9;FCER1G;CX3CL1;NPPB;STAR;MAPK8IP3;SH3KBP1;NUP210;NFKBIZ;ABCG1;ATF3;CCL7;PRG4;GJA5;RASD1;FOXK2;NR4A3;PTX3;NOS2;BDKRB1;CYP7B1;CFLAR;TFPI2;MMP3;IL6;SDCBP2;FANCD2;RNASE1;HPGD;ELN;CXCL1;SH3BP4;TERC;ZC3H12A;SLC4A11;C3;MMP13;EGLN3;PTGES;LCN2;FGF9;BAALC;CILP;PDE1A;SLPI;TSC22D3;SERPINE1;CYP26B1;DDIT3;HP;PLA2G2A;Tmeff2;MT2A
6	**apoptosis**	**1.34007E-13**	**SPRY1;SELP;G0S2;CXCL2;CXCL5;STEAP4;TFRC;CXCL3;DUSP5;CD180;RGS2;BANP;SMPD3;C1QTNF3;BTG2;TGM1;TNFRSF9;GALNT14;SNAI2;NR4A1;TLR2;TNFRSF12A;FCER1G;MMP9;CX3CL1;PLN;NPPB;STAR;MAPK8IP3;SH3KBP1;HSPA1B;NUP210;NFKBIZ;ABCG1;ATF3;CCL7;PRG4;GJA5;RASD1;NR4A3;PTX3;NOS2;BDKRB1;CYP7B1;CFLAR;MT1A;TFPI2;MMP3;IL6;SDCBP2;PPM1E;FANCD2;HPGD;ELN;CXCL1;TERC;ZC3H12A;SLC4A11;C3;MMP13;EGLN3;PTGES;LCN2;PCP4;FGF9;BAALC;PDE1A;SLPI;TSC22D3;SERPINE1;CYP26B1;DDIT3;HP;PLA2G2A;Tmeff2;MT2A**
7	cell differentiation	2.32307E-13	SPRY1;SELP;G0S2;OGN;CXCL2;APOC2;CXCL5;STEAP4;TFRC;H2AFY2;DUSP5;CD180;RGS2;BANP;SMPD3;C1QTNF3;FLRT3;BTG2;TGM1;TNFRSF9;SLC5A3;UAP1;SNAI2;NR4A1;TLR2;TNFRSF12A;FCER1G;MMP9;CX3CL1;NPPB;STAR;MAPK8IP3;SH3KBP1;NUP210;NFKBIZ;ABCG1;ATF3;CCL7;GJA5;RASD1;NR4A3;PTX3;NOS2;BDKRB1;PDE8A;CFLAR;TFPI2;MMP3;IL6;FANCD2;HPGD;ELN;CXCL1;TERC;ZC3H12A;C3;MMP13;EGLN3;PTGES;LCN2;PCP4;FGF9;BAALC;SLPI;TSC22D3;SERPINE1;CYP26B1;DDIT3;HP;PLA2G2A;Tmeff2;TNIP3;MT2A
8	wound healing	4.50471E-13	SPRY1;NOS2;BDKRB1;SELP;TFPI2;MMP3;IL6;CXCL2;ELN;CXCL1;TGM1;C3;MMP13;PTGES;LAMC2;SNAI2;LCN2;TLR2;TNFRSF12A;FGF9;MMP9;CX3CL1;SLPI;NPPB;SERPINE1;HP;CCL7;PTX3;MT2A
9	leukocyte migration	6.00704E-13	NOS2;LCN2;BDKRB1;SELP;TLR2;MMP3;MMP9;IL6;CXCL2;CX3CL1;NPPB;CXCL5;SERPINE1;ELN;CXCL1;CXCL3;ATF3;ABCG1;CCL7;MMP13;TNFRSF9;PTX3
10	neutrophil recruitment	6.47297E-13	NOS2;LCN2;BDKRB1;SELP;MMP3;TLR2;TNFRSF12A;IL6;MMP9;CXCL2;CX3CL1;SLPI;CXCL5;SERPINE1;CXCL1;CXCL3;CCL7;PTX3
11	vascularization	1.46597E-12	NOS2;SPRY1;BDKRB1;CFLAR;SELP;TFPI2;MMP3;IL6;OGN;CXCL2;CXCL5;HPGD;RNASE1;ELN;CXCL1;TERC;TFRC;CXCL3;ZC3H12A;SMPD3;C1QTNF3;C3;MMP13;EGLN3;PTGES;LAMC2;LCN2;NR4A1;TLR2;TNFRSF12A;FGF9;MMP9;CX3CL1;NPPB;SERPINE1;CYP26B1;DDIT3;HP;ATF3;PLA2G2A;CCL7;PTX3;MT2A
12	cell migration	1.88498E-12	RND1;SPRY1;SELP;CXCL2;STEAP4;CXCL5;CXCL3;DUSP5;BANP;SMPD3;FLRT3;BTG2;TNFRSF9;GALNT14;LAMC2;SNAI2;EHBP1;NR4A1;TLR2;TNFRSF12A;MMP9;CX3CL1;NPPB;SCGB1C1;MAPK8IP3;SH3KBP1;ATF3;CCL7;GJA5;PTX3;NOS2;BDKRB1;TFPI2;MMP3;IL6;HPGD;ELN;CXCL1;SH3BP4;C3;EGLN3;MMP13;PTGES;LCN2;FGF9;SLPI;TSC22D3;SERPINE1;DDIT3;HP;Tmeff2;PLA2G2A
13	ROS generation	2.79604E-12	NOS2;BDKRB1;SELP;MMP3;IL6;OGN;CXCL2;APOC2;STEAP4;ELN;CXCL1;TFRC;ZC3H12A;C1QTNF3;BTG2;TNFRSF9;PTGES;LCN2;TLR2;FGF9;FCER1G;MMP9;CX3CL1;SLPI;NPPB;SERPINE1;DDIT3;HP;ATF3;ABCG1;PLA2G2A;GJA5;NR4A3;PTX3;MT2A
14	cell survival	2.91509E-12	CXCL2;CXCL5;TFRC;DUSP5;BTG2;TNFRSF9;SNAI2;UAP1;NR4A1;TNFRSF12A;TLR2;FCER1G;MMP9;PLN;CX3CL1;NPPB;SH3KBP1;HSPA1B;NFKBIZ;ATF3;ABCG1;CCL7;NR4A3;NOS2;CYP7B1;CFLAR;TFPI2;MMP3;IL6;SDCBP2;FANCD2;ELN;CXCL1;SH3BP4;ZC3H12A;SLC4A11;C3;EGLN3;MMP13;PTGES;LCN2;FGF9;TSC22D3;SERPINE1;CYP26B1;DDIT3;HP;Tmeff2;MT2A
15	calcium mobilization	8.43475E-12	NOS2;SPRY1;CFLAR;SELP;TLR2;FGF9;FCER1G;IL6;MMP9;CXCL2;CX3CL1;PLN;CXCL5;CXCL1;SH3KBP1;TFRC;CXCL3;CD180;RGS2;HP;PLA2G2A;CCL7;C3;NR4A3
16	innate immune response	2.12494E-11	NOS2;LCN2;NR4A1;CFLAR;TLR2;IL6;CXCL2;CX3CL1;SLPI;CXCL5;SERPINE1;ELN;ZC3H12A;NFKBIZ;CD180;HP;ATF3;ABCG1;PLA2G2A;C3;TNFRSF9;PTX3
17	tissue remodeling	3.20709E-11	NOS2;LCN2;CFLAR;SELP;TFPI2;MMP3;TNFRSF12A;MMP9;IL6;CXCL2;CX3CL1;SLPI;CXCL5;SERPINE1;ELN;HP;MMP13;PTX3
18	**cell death**	**3.54918E-11**	**SPRY1;CXCL2;STEAP4;TFRC;CD180;RGS2;BANP;SMPD3;FLRT3;TGM1;BTG2;TNFRSF9;SNAI2;NR4A1;TLR2;TNFRSF12A;FCER1G;MMP9;CX3CL1;PLN;NPPB;SH3KBP1;NUP210;NFKBIZ;ATF3;ABCG1;CCL7;RASD1;GJA5;NR4A3;PTX3;NOS2;BDKRB1;CFLAR;TFPI2;MMP3;IL6;SDCBP2;PPM1E;FANCD2;HPGD;RNASE1;ELN;CXCL1;ZC3H12A;EGLN3;PTGES;LCN2;PCP4;FGF9;TSC22D3;SERPINE1;DDIT3;PLA2G2A;MT2A**
19	blood vessel permeability	8.41257E-11	LAMC2;PTGES;NOS2;BDKRB1;LCN2;NR4A1;MMP3;TLR2;TNFRSF12A;MMP9;IL6;CXCL2;SLPI;NPPB;SERPINE1;ELN;CXCL1;C3;PTX3
20	leukocyte recruitment	8.46938E-11	NOS2;BDKRB1;LCN2;SELP;TLR2;IL6;MMP9;CXCL2;CX3CL1;TSC22D3;CXCL1;CXCL3;CCL7;GJA5;TNFRSF9;PTX3
21	microglial activation	9.65511E-11	NOS2;LCN2;BDKRB1;SELP;MMP3;TLR2;FCER1G;MMP9;IL6;CX3CL1;SLPI;NPPB;SERPINE1;CXCL1;ZC3H12A;PLA2G2A;C3;MT2A;PTX3
22	NO biosynthesis	1.23107E-10	PTGES;NOS2;BDKRB1;LCN2;SELP;MMP3;TLR2;IL6;CXCL2;CX3CL1;SLPI;ELN;DDIT3;ZC3H12A;SMPD3;ABCG1;ATF3;GJA5
23	pregnancy	2.6908E-10	RND1;NOS2;MT1A;SELP;TFPI2;MMP3;IL6;CXCL2;HPGD;ELN;TFRC;RGS2;TNFRSF9;PTGES;LCN2;NR4A1;PCP4;TLR2;MMP9;CX3CL1;SLPI;NPPB;SERPINE1;STAR;HP;ATF3;ABCG1;GJA5;PTX3;MT2A
24	**calcium ion homeostasis**	**2.73819E-10**	**NOS2;BDKRB1;CFLAR;SELP;IL6;CXCL2;PLCL1;CXCL5;ELN;CXCL1;CD180;RGS2;MMP13;TNFRSF9;NR4A1;PCP4;TLR2;FGF9;FCER1G;MMP9;PLN;CX3CL1;NPPB;TSC22D3;SERPINE1;DDIT3;CCL7;PLA2G2A;RASD1;GJA5**
25	monocyte recruitment	4.30453E-10	NR4A1;SELP;TLR2;IL6;MMP9;CXCL2;CX3CL1;CXCL5;ELN;CXCL1;CCL7;NR4A3;TNFRSF9
26	immunity	5.95146E-10	NOS2;CFLAR;SELP;MMP3;IL6;CXCL2;CXCL5;ELN;CXCL1;TFRC;ZC3H12A;BTG2;C3;TNFRSF9;PTGES;LCN2;TLR2;TNFRSF12A;MMP9;CX3CL1;SLPI;TSC22D3;SERPINE1;Fv1;NFKBIZ;HP;ATF3;ABCG1;CCL7;PLA2G2A;PTX3
27	cytokine production	6.88583E-10	NOS2;BDKRB1;CFLAR;SELP;MMP3;IL6;ELN;TFRC;ZC3H12A;CD180;C1QTNF3;TNFRSF9;PTGES;LCN2;NR4A1;PCP4;TLR2;TNFRSF12A;FCER1G;CX3CL1;SLPI;DDIT3;ATF3;CCL7;PLA2G2A;GJA5;PTX3
28	heart function	8.50623E-10	PTGES;NOS2;BDKRB1;LCN2;TLR2;MMP9;IL6;CX3CL1;PLN;SLPI;NPPB;SERPINE1;ELN;CXCL1;CXCL3;CD180;RGS2;ATF3;C1QTNF3;GJA5;MMP13;EGLN3;PTX3
29	blood clotting	1.08924E-09	PTGES;NOS2;SELP;TFPI2;TLR2;MMP3;FCER1G;MMP9;IL6;CX3CL1;SLPI;NPPB;TSC22D3;SERPINE1;ELN;NUP210;HP;PLA2G2A;TGM1;C3;PTX3
30	immunoreactivity	1.47715E-09	LAMC2;NOS2;LCN2;SELP;TLR2;MMP3;IL6;MMP9;PDE1A;CX3CL1;SLPI;NPPB;TSC22D3;DDIT3;TFRC;ATF3;CCL7;C3;NR4A3;TNFRSF9
31	degranulation	1.51232E-09	NOS2;SELP;TLR2;FCER1G;IL6;MMP9;CXCL2;CX3CL1;SLPI;CXCL5;ELN;CXCL1;SH3KBP1;CCL7;PLA2G2A;C3;NR4A3;TNFRSF9
32	kidney function	2.26454E-09	PTGES;NOS2;LCN2;BDKRB1;NR4A1;SELP;MT1A;TLR2;TNFRSF12A;MMP9;IL6;NPPB;SERPINE1;DDIT3;CD180;HP;PLA2G2A
33	endothelial cell proliferation	2.74262E-09	SPRY1;LCN2;BDKRB1;NR4A1;TFPI2;TLR2;MMP3;FGF9;MMP9;IL6;CXCL2;CX3CL1;CXCL5;SERPINE1;ELN;CXCL1;C1QTNF3;NR4A3;TNFRSF9;PTX3
34	ossification	4.27153E-09	PTGES;NOS2;SPRY1;SLC5A3;MMP3;FGF9;OGN;MMP9;IL6;CX3CL1;NPPB;SERPINE1;ELN;CYP26B1;CXCL1;DDIT3;ZC3H12A;RGS2;SMPD3;CCL7;PRG4;C3;MMP13
35	neutrophil function	4.92221E-09	NOS2;CXCL1;LCN2;HP;TLR2;IL6;MMP9;CXCL2;SLPI;EGLN3;CXCL5
36	neuronal death	5.31247E-09	PTGES;NOS2;BDKRB1;LCN2;NR4A1;PCP4;TLR2;TNFRSF12A;FGF9;MMP3;FCER1G;IL6;MMP9;CXCL2;CX3CL1;SERPINE1;CXCL1;DDIT3;ATF3;PLA2G2A;RASD1;C3;EGLN3;MT2A;PTX3
37	luteinization	7.14186E-09	NOS2;SNAI2;NR4A1;TNFRSF12A;MMP3;IL6;MMP9;CX3CL1;SERPINE1;CXCL1;STAR;BTG2
38	T lymphocyte proliferation	7.67743E-09	NOS2;LCN2;NR4A1;CFLAR;TLR2;TNFRSF12A;MMP9;IL6;CX3CL1;TSC22D3;SERPINE1;DDIT3;TFRC;CXCL3;DUSP5;RGS2;HP;PLA2G2A;BTG2;C3;TNFRSF9
39	monocyte adhesion	1.10535E-08	NR4A1;SELP;TLR2;IL6;CX3CL1;SERPINE1;ELN;CXCL1;ZC3H12A;ABCG1;GJA5;C3;NR4A3;TNFRSF9
40	neutrophil adhesion	1.23666E-08	NOS2;BDKRB1;SELP;IL6;CXCL2;CX3CL1;CXCL5;CXCL1;HP;PLA2G2A;GJA5;PTX3
41	positive chemotaxis	1.42655E-08	ELN;CXCL1;TFPI2;MMP3;IL6;MMP9;CXCL2;CX3CL1;MMP13;CXCL5;TNFRSF9
42	regeneration	1.7138E-08	SPRY1;NOS2;MMP3;OGN;IL6;CXCL2;ELN;CXCL1;TERC;CXCL3;C1QTNF3;TGM1;C3;MMP13;LCN2;TLR2;FGF9;TNFRSF12A;MMP9;SLPI;NPPB;SERPINE1;DDIT3;HP;ATF3;CCL7;GJA5;MT2A
43	adipocyte differentiation	2.24277E-08	PTGES;SPRY1;LCN2;NR4A1;EHBP1;MMP3;G0S2;IL6;MMP9;TSC22D3;STEAP4;SERPINE1;DDIT3;RGS2;ATF3;C3;EGLN3;NR4A3;MT2A
44	lymphocyte proliferation	2.94726E-08	NOS2;CFLAR;G0S2;IL6;MMP9;CXCL2;SLPI;NPPB;TFRC;CD180;HP;ABCG1;TNFRSF9;PTX3
45	oxidative stress	3.02872E-08	NOS2;BDKRB1;CFLAR;SELP;MT1A;MMP3;IL6;CXCL2;RNASE1;ELN;CXCL1;TFRC;ZC3H12A;SMPD3;BTG2;PTGES;LCN2;TLR2;FCER1G;MMP9;SLPI;NPPB;SERPINE1;DDIT3;SH3KBP1;HP;ATF3;ABCG1;PLA2G2A;GJA5;NR4A3;MT2A

Table S1-2. Cell processes significantly related to the 91 DEGs in RASMC treated with

CMS

	cell process	p-value	Overlapping Entities
46	artery remodeling	3.12413E-08	SERPINE1;NOS2;ELN;TFRC;NR4A1;CD180;MMP3;TLR2;IL6;MMP9
47	endothelial cell activation	4.30498E-08	SERPINE1;NOS2;NR4A1;ZC3H12A;SELP;TFPI2;TLR2;IL6;GJA5;CX3CL1;TNFRSF9
48	ovulation	4.58624E-08	NOS2;NR4A1;TNFRSF12A;MMP3;TLR2;IL6;MMP9;CX3CL1;SERPINE1;HPGD;CXCL1;STAR;MMP13;PTX3
49	granulocyte production	5.792E-08	NOS2;CXCL1;BDKRB1;SELP;IL6;CXCL2;CXCL5
50	chemotaxis	6.03229E-08	NOS2;BDKRB1;NR4A1;PDE8A;SELP;TLR2;MMP9;IL6;CXCL2;CX3CL1;NPPB;CXCL5;SERPINE1;ELN;CXCL1;CXCL3;RGS2;HP;ABCG1;CCL7;C3;MMP13;PTX3
51	T-cell recruitment	7.12144E-08	NOS2;CXCL1;LCN2;BDKRB1;SELP;TLR2;IL6;MMP9;CX3CL1
52	cell adhesion	8.30389E-08	RND1;NOS2;PDE8A;SELP;TFPI2;MMP3;OGN;IL6;CXCL2;HPGD;ELN;CXCL1;TFRC;ZC3H12A;SMPD3;FLRT3;MMP13;TNFRSF9;LAMC2;SNAI2;TLR2;MMP9;CX3CL1;TSC22D3;SERPINE1;SH3KBP1;MAPK8IP3;ATF3;PRG4;PLA2G2A;Tmeff2;GJA5
53	contraction	1.04635E-07	RND1;NOS2;BDKRB1;PDE8A;SELP;MMP3;IL6;HPGD;ELN;RGS2;C3;MMP13;TNFRSF9;LCN2;PCP4;TLR2;MMP9;PLN;CX3CL1;PDE1A;SLPI;NPPB;SERPINE1;CCL7;GJA5;PTX3
54	epithelial cell proliferation	1.36333E-07	NOS2;SPRY1;CFLAR;FGF9;MMP3;TLR2;IL6;MMP9;CXCL2;SDCBP2;SLPI;SERPINE1;HPGD;DUSP5;SMPD3
55	lipid metabolism	1.58674E-07	NOS2;LCN2;NR4A1;TLR2;G0S2;IL6;APOC2;CX3CL1;NPPB;STEAP4;SERPINE1;DDIT3;STAR;HP;ABCG1;C1QTNF3;PLA2G2A;C3;MT2A;TNFRSF9;PTX3
56	neuroprotection	1.98402E-07	NOS2;BDKRB1;NR4A1;PCP4;FGF9;IL6;MMP9;CXCL2;CX3CL1;SLPI;SERPINE1;CXCL1;DDIT3;STAR;RGS2;ATF3;BTG2;MT2A
57	apoptosis of neutrophils	2.00269E-07	SERPINE1;NOS2;CXCL1;LCN2;TLR2;IL6;CXCL2;SLPI;EGLN3;TNFRSF9
58	neutrophil chemotaxis	2.28339E-07	NOS2;ELN;CXCL1;BDKRB1;CXCL3;HP;IL6;MMP9;CXCL2;SLPI;CXCL5
59	hemato-encephalic barrier	2.70191E-07	NOS2;BDKRB1;SELP;MMP3;TLR2;IL6;MMP9;CXCL2;CX3CL1;SERPINE1;ELN;CXCL1;TFRC;MMP13
60	vasoconstriction	2.77878E-07	NOS2;LCN2;BDKRB1;SELP;TLR2;IL6;MMP9;CX3CL1;NPPB;SERPINE1;HPGD;RGS2;SMPD3;GJA5
61	fibrinolysis	3.6987E-07	SERPINE1;ELN;SELP;TFPI2;MMP3;IL6;MMP9;C3;MMP13;PTX3
62	blood circulation	3.78755E-07	NOS2;BDKRB1;LCN2;SELP;FGF9;MMP9;IL6;CX3CL1;NPPB;SERPINE1;ELN;CXCL1;CD180;HP;GJA5;C3
63	fertilization	4.0383E-07	LCN2;SELP;TFPI2;TLR2;MMP9;IL6;PDE1A;CX3CL1;FANCD2;NPPB;SH3KBP1;STAR;HP;ABCG1;CCL7;MMP13;PTX3
64	neuron apoptoses	4.25003E-07	SERPINE1;NOS2;DDIT3;TFRC;TLR2;FGF9;ATF3;IL6;PLA2G2A;MMP9;CX3CL1;EGLN3
65	lipid peroxidation	4.28573E-07	NOS2;SELP;IL6;MMP9;CXCL2;NPPB;TSC22D3;DDIT3;TFRC;HP;PLA2G2A;C3;MT2A
66	immune cell chemotaxis	5.54041E-07	ELN;CXCL1;CXCL3;CCL7;IL6;CXCL2;CX3CL1;CXCL5
67	leukocyte cell adhesion	5.71692E-07	NOS2;CXCL1;SELP;TLR2;IL6;MMP9;GJA5;CXCL2;CX3CL1;SLPI;MMP13;PTX3
68	neutrophil activation	6.0091E-07	SERPINE1;CXCL1;CXCL3;SELP;TLR2;IL6;MMP9;CXCL2;SLPI;CXCL5
69	macrophage activation	6.06922E-07	NOS2;NR4A1;TLR2;FCER1G;IL6;CX3CL1;SLPI;CXCL5;ZC3H12A;CD180;HP;ATF3;NR4A3;TNFRSF9
70	DNA replication	6.94572E-07	SPRY1;NOS2;BDKRB1;CFLAR;TFPI2;IL6;FANCD2;CXCL5;CXCL1;TFRC;TERC;ZC3H12A;CD180;SMPD3;C1QTNF3;C3;PTGES;LCN2;NR4A1;TLR2;TNFRSF12A;FGF9;SLPI;NPPB;DDIT3;ATF3;NR4A3;PTX3
71	leukocyte tethering / rolling	7.13445E-07	NOS2;CXCL1;SELP;TLR2;MMP9;CXCL2;CX3CL1;SLPI
72	nerve regeneration	7.77224E-07	NOS2;MMP3;TLR2;ATF3;TNFRSF12A;IL6;MMP9;RASD1;FLRT3;CX3CL1;MT2A
73	leukocyte accumulation	8.57338E-07	CXCL1;BDKRB1;SELP;TLR2;IL6;MMP9;CX3CL1;SLPI
74	cell invasion	9.01605E-07	NOS2;BDKRB1;SLC16A3;TFPI2;MMP3;IL6;ELN;CXCL1;EGLN3;MMP13;GALNT14;PTGES;LAMC2;SNAI2;LCN2;NR4A1;TLR2;FGF9;TNFRSF12A;MMP9;CX3CL1;SLPI;TSC22D3;SERPINE1;ATF3;PLA2G2A;MT2A
75	respiratory burst	1.15952E-06	NOS2;CXCL1;SELP;HP;TLR2;CCL7;FCER1G;IL6;CXCL2;CXCL5
76	tissue regeneration	1.30397E-06	SERPINE1;ELN;SNAI2;MMP3;TLR2;TNFRSF12A;CCL7;IL6;PRG4;MT2A
77	endothelial cell function	1.32035E-06	NOS2;SPRY1;PDE8A;SELP;TFPI2;TLR2;IL6;MMP9;CX3CL1;SERPINE1;ELN;CXCL1;DDIT3;ABCG1;PTX3
78	luteolysis	1.36771E-06	SERPINE1;NOS2;STAR;RGS2;ATF3;PLA2G2A;MMP9;MMP13
79	tissue maintenance	1.54163E-06	NOS2;CFLAR;HP;FGF9;MMP3;TLR2;TNFRSF12A;IL6;MMP9;MMP13;PTX3
80	platelet activation	1.91824E-06	SERPINE1;ELN;NOS2;SELP;MMP3;TLR2;FCER1G;MMP9;IL6;CX3CL1;SLPI;CXCL5
81	bone resorption	2.06992E-06	PTGES;NOS2;SPRY1;TLR2;MMP3;FCER1G;IL6;MMP9;CXCL2;CX3CL1;NPPB;TSC22D3;SERPINE1;HP;MMP13
82	arteriogenesis	2.60725E-06	NOS2;ELN;BDKRB1;TLR2;MMP9;GJA5;NPPB;EGLN3
83	parturition	2.83307E-06	PTGES;HPGD;NOS2;LCN2;HP;TLR2;IL6;PLA2G2A;MMP9;MMP13
84	cardiovascular function	3.08198E-06	SERPINE1;NOS2;ELN;BDKRB1;RGS2;IL6;GJA5;CX3CL1;NPPB
85	digestion	3.40905E-06	TFPI2;TLR2;MMP3;IL6;MMP9;APOC2;SLPI;RNASE1;ELN;HP;PLA2G2A;C3;MMP13
86	myocyte function	4.27176E-06	SERPINE1;ELN;SLC16A3;IL6;MMP9;PLN;NPPB;NR4A3
87	Glucose metabolism	5.06705E-06	NOS2;LCN2;NR4A1;IL6;APOC2;CX3CL1;NPPB;SERPINE1;DDIT3;TFRC;ATF3;C1QTNF3;BTG2;C3;EGLN3;TNFRSF9;PTX3
88	phagocyte activity	5.20394E-06	SERPINE1;ELN;NOS2;LCN2;HP;TLR2;IL6;CX3CL1;C3;PTX3
89	cholesterol export	5.37527E-06	NOS2;LCN2;STAR;HP;ABCG1;IL6;MMP9;APOC2;CXCL5;PTX3
90	embryonal development	5.57656E-06	SPRY1;RND1;NOS2;CYP7B1;CFLAR;SELP;MMP3;IL6;FANCD2;ELN;TFRC;RGS2;FLRT3;BTG2;C3;MMP13;SNAI2;NR4A1;PCP4;TLR2;TNFRSF12A;FGF9;FCER1G;MMP9;NPPB;SERPINE1;CYP26B1;STAR;MAPK8IP3;HP;ABCG1;PTX3;MT2A
91	cell cycle	5.9583E-06	NOS2;CFLAR;SELP;TFPI2;IL6;G0S2;FANCD2;STEAP4;HPGD;ELN;CXCL1;TERC;TFRC;DUSP5;BANP;SMPD3;BTG2;MMP13;EGLN3;TNFRSF9;PTGES;LAMC2;SNAI2;LCN2;UAP1;NR4A1;TNFRSF12A;FGF9;MMP9;PDE1A;CX3CL1;SLPI;TSC22D3;SERPINE1;CYP26B1;DDIT3;MAPK8IP3;STAR;ATF3;ABCG1;Tmeff2;CCL7;FOXK2;NR4A3;MT2A
92	bone remodeling	7.0992E-06	SERPINE1;NOS2;SLC5A3;DDIT3;CXCL3;TLR2;MMP3;FCER1G;IL6;PLA2G2A;MMP9;MMP13
93	Glycan catabolism	8.61465E-06	NOS2;MMP3;IL6;MMP9;MMP13
94	relaxation	9.03156E-06	PTGES;NOS2;BDKRB1;SELP;MMP9;IL6;CX3CL1;PLN;NPPB;ELN;DDIT3;RGS2;HP;ABCG1;PLA2G2A;NR4A3
95	osteoclast differentiation	9.36021E-06	NOS2;TNFRSF12A;TLR2;MMP9;IL6;FCER1G;CXCL2;CX3CL1;STEAP4;CXCL1;BTG2;MMP13;TNFRSF9
96	cartilage homeostasis	1.08606E-05	MMP3;IL6;PRG4;MMP9;CILP;MMP13
97	response to stimulus	1.15778E-05	CXCL1;CXCL3;IL6;CXCL2
98	T-cell development	1.19398E-05	NOS2;NR4A1;CFLAR;FGF9;TLR2;FCER1G;IL6;CXCL2;TSC22D3;TFRC;CXCL3;DUSP5;CD180;BANP;NR4A3;TNFRSF9
99	reproductive process	1.3299E-05	PTGES;SERPINE1;HP;IL6;MMP9;NPPB
100	glucose homeostasis	1.43877E-05	NOS2;LCN2;NR4A1;CYP7B1;TLR2;G0S2;IL6;SLC45A1;STEAP4;DDIT3;HP;ATF3;BTG2;EGLN3

Table S2-1. Enrichment analysis of the DEGs focusing on "Diseases"

	Gene Set Seed	p-value	Overlapping Entities
1	Atherosclerosis	1.95486E-26	NOS2;BDKRB1;CYP7B1;SELP;TFPI2;SLC16A3;MMP3;IL6;OGN;CXCL2;APOC2;CXCL5;STEAP4;ELN;CXCL1;CXCL3;ZC3H12A;CD180;RGS2;C3;MMP13;TNFRSF9;PTGES;LCN2;NR4A1;TLR2;TNFRSF12A;MMP9;PDE1A;CX3CL1;NPPB;SERPINE1;CYP26B1;DDIT3;STAR;NFKBIZ;HP;ATF3;ABCG1;PLA2G2A;CCL7;GJA5;NR4A3;PTX3;MT2A
2	Wounds and Injuries	1.01661E-20	NOS2;BDKRB1;CFLAR;SELP;MT1A;TFPI2;MMP3;IL6;CXCL2;CXCL5;HPGD;ELN;CXCL1;TFRC;CXCL3;RGS2;FLRT3;TGM1;C3;MMP13;EGLN3;TNFRSF9;PTGES;LCN2;NR4A1;TLR2;TNFRSF12A;MMP9;FCER1G;CX3CL1;PLN;CILP;PDE1A;SLPI;NPPB;TSC22D3;SERPINE1;STAR;DDIT3;HSPA1B;HP;ABCG1;ATF3;CCL7;PRG4;PLA2G2A;GJA5;NR4A3;PTX3;MT2A
3	Inflammation	2.7854E-19	SPRY1;SELP;CXCL2;APOC2;STEAP4;CXCL5;TFRC;CXCL3;CD180;RGS2;SMPD3;C1QTNF3;TNFRSF9;SNAI2;NR4A1;TLR2;TNFRSF12A;FCER1G;MMP9;CX3CL1;NPPB;STAR;NFKBIZ;ATF3;ABCG1;PRG4;CCL7;GJA5;PTX3;NOS2;BDKRB1;CYP7B1;TFPI2;MMP3;IL6;HPGD;ELN;CXCL1;ZC3H12A;C3;EGLN3;MMP13;PTGES;LCN2;SLPI;TSC22D3;SERPINE1;CYP26B1;DDIT3;HP;PLA2G2A;MT2A
4	Fibrosis	5.51182E-19	NOS2;BDKRB1;SELP;MMP3;OGN;IL6;CXCL2;CXCL5;ELN;CXCL1;RGS2;C1QTNF3;EGLN3;MMP13;PTGES;SNAI2;LCN2;TLR2;TNFRSF12A;FCER1G;MMP9;PDE1A;CX3CL1;PLN;SLPI;NPPB;SERPINE1;DDIT3;STAR;HP;ATF3;PRG4;PLA2G2A;CCL7;MT2A
5	Acute Lung Injury	3.04134E-16	NOS2;LCN2;BDKRB1;SELP;TFPI2;TLR2;MMP3;MMP9;IL6;CXCL2;CX3CL1;SLPI;NPPB;CXCL5;SERPINE1;ELN;CXCL1;ATF3;PLA2G2A;EGLN3;MMP13;PTX3
6	Pulmonary Fibrosis	1.34592E-15	PTGES;NOS2;SPRY1;LCN2;SELP;TLR2;MMP3;FGF9;MMP9;IL6;CXCL2;CX3CL1;SLPI;NPPB;CXCL5;SERPINE1;ELN;DDIT3;TERC;CXCL3;CCL7;C1QTNF3;GJA5;MMP13
7	Stroke	7.25809E-15	NOS2;BDKRB1;SELP;MMP3;IL6;CXCL2;RNASE1;ELN;CXCL1;ZC3H12A;C3;PTGES;LCN2;TLR2;TNFRSF12A;MMP9;PLN;CX3CL1;SLPI;NPPB;SERPINE1;HP;ATF3;CCL7;PLA2G2A;PTX3
8	Arthritis	9.80339E-15	PTGES;NOS2;SELP;TLR2;MMP3;FCER1G;IL6;MMP9;CXCL2;CILP;CX3CL1;SLPI;CXCL5;STEAP4;TSC22D3;SERPINE1;CXCL1;DUSP5;HP;PLA2G2A;C1QTNF3;C3;MMP13;TNFRSF9;PTX3
9	Fatty Streak, Arterial	2.40459E-14	PTGES;NOS2;LCN2;SELP;TFPI2;TLR2;MMP9;IL6;CX3CL1;SLPI;SERPINE1;ELN;DDIT3;CD180;HP;CCL7;MMP13;TNFRSF9;PTX3
10	Infarction	2.67115E-14	NOS2;BDKRB1;LCN2;SELP;TLR2;TNFRSF12A;IL6;MMP9;CXCL2;CX3CL1;PLN;SLPI;NPPB;SERPINE1;ELN;CXCL1;DDIT3;ZC3H12A;ATF3;PLA2G2A;C1QTNF3;BTG2;MMP13;EGLN3;PTX3
11	Arthritis, Rheumatoid	9.84104E-14	NOS2;BDKRB1;SELP;MMP3;IL6;CXCL2;CXCL5;STEAP4;CXCL1;TFRC;C3;MMP13;TNFRSF9;PTGES;LCN2;TNFRSF12A;TLR2;MMP9;CX3CL1;TSC22D3;SERPINE1;DDIT3;PRG4;PLA2G2A;PTX3
12	leukocyte infiltration	1.02535E-13	PTGES;NOS2;LCN2;NR4A1;SELP;MMP3;TLR2;TNFRSF12A;IL6;MMP9;CXCL2;CX3CL1;SLPI;CXCL1;ATF3;CCL7;MT2A;TNFRSF9
13	kidney toxicity	2.96018E-13	PTGES;NOS2;BDKRB1;LCN2;NR4A1;CFLAR;MT1A;SELP;TLR2;TNFRSF12A;MMP9;IL6;CXCL2;CX3CL1;NPPB;SERPINE1;CXCL1;DDIT3;HP;ATF3;CCL7;MT2A;PTX3
14	Sepsis	3.32169E-13	NOS2;LCN2;BDKRB1;NR4A1;PCP4;SELP;TLR2;FCER1G;MMP9;IL6;CXCL2;NPPB;SERPINE1;DDIT3;ZC3H12A;ATF3;PLA2G2A;GJA5;C3;MMP13;TNFRSF9;PTX3
15	vascular remodeling	5.10398E-13	NOS2;LCN2;NR4A1;SELP;MMP3;TNFRSF12A;MMP9;IL6;CX3CL1;NPPB;SERPINE1;HPGD;ELN;DDIT3;TFRC;STAR;RGS2;HP;C1QTNF3;GJA5;C3;MMP13
16	macrophage infiltration	5.51041E-13	NOS2;SPRY1;BDKRB1;SELP;MMP3;TLR2;TNFRSF12A;IL6;MMP9;CXCL2;CX3CL1;SERPINE1;CXCL1;DDIT3;ABCG1;CCL7;C3;TNFRSF9
17	Extravasation of Diagnostic and Therapeutic Materials	5.7636E-13	NOS2;LCN2;BDKRB1;SELP;MMP3;TLR2;IL6;MMP9;CXCL2;CX3CL1;NPPB;CXCL5;SERPINE1;CXCL1;CXCL3;CCL7;C3;TNFRSF9;PTX3
18	Obesity	6.1146E-13	SPRY1;NOS2;BDKRB1;MMP3;IL6;STEAP4;ELN;CXCL1;TFRC;CD180;C3;MMP13;TNFRSF9;LCN2;NR4A1;TLR2;TNFRSF12A;MMP9;CX3CL1;NPPB;SERPINE1;DDIT3;HP;ATF3;ABCG1;PLA2G2A;RASD1;PTX3;MT2A
19	heart remodeling	6.73492E-13	NOS2;BDKRB1;LCN2;NR4A1;MMP3;TLR2;TNFRSF12A;MMP9;IL6;PDE1A;PLN;NPPB;SERPINE1;ELN;CD180;ATF3;CCL7;C1QTNF3;GJA5;C3;MMP13
20	Ischemia	8.16101E-13	NOS2;BDKRB1;SELP;MMP3;IL6;CXCL2;CXCL5;ELN;CXCL1;ZC3H12A;CD180;C1QTNF3;PTGES;LCN2;NR4A1;TLR2;MMP9;PLN;CX3CL1;SLPI;NPPB;SERPINE1;DDIT3;MAPK8IP3;HP;PLA2G2A;NR4A3;MT2A
21	Myocardial Infarction	3.39473E-12	PTGES;NOS2;SELP;TLR2;MMP3;TNFRSF12A;FGF9;MMP9;IL6;CX3CL1;PLN;SLPI;NPPB;SERPINE1;ELN;CXCL1;DDIT3;CD180;HP;CCL7;C1QTNF3;MMP13;PTX3
22	Neoplasms	4.37875E-12	SPRY1;SELP;OGN;G0S2;CXCL2;CXCL5;STEAP4;TFRC;CXCL3;RGS2;BANP;SMPD3;C1QTNF3;BTG2;TGM1;TNFRSF9;LAMC2;MLH3;SNAI2;NR4A1;TLR2;TNFRSF12A;FCER1G;MMP9;CX3CL1;SH3KBP1;STAR;NFKBIZ;ATF3;CCL7;RASD1;NR4A3;PTX3;NOS2;BDKRB1;CFLAR;MT1A;TFPI2;SLC16A3;MMP3;IL6;FANCD2;HPGD;RNASE1;CXCL1;TERC;C3;MMP13;EGLN3;PTGES;LCN2;FGF9;SLPI;TSC22D3;SERPINE1;DDIT3;Fv1;HP;PLA2G2A;Tmeff2;TNIP3;MT2A
23	Endometriosis	4.90823E-12	LAMC2;PTGES;NOS2;LCN2;SNAI2;MMP3;FGF9;IL6;MMP9;SLPI;CXCL5;SERPINE1;HPGD;CXCL1;STAR;HP;C3
24	Insulin Resistance	5.45539E-12	NOS2;BDKRB1;SELP;MMP3;G0S2;IL6;STEAP4;CXCL5;ELN;DUSP5;CD180;C1QTNF3;C3;TNFRSF9;LCN2;NR4A1;TLR2;CX3CL1;SLPI;NPPB;SERPINE1;DDIT3;ABCG1;ATF3;NR4A3;PTX3;MT2A
25	Lung Injury	6.08454E-12	NOS2;SELP;TLR2;MMP3;IL6;MMP9;CXCL2;SLPI;CXCL5;SERPINE1;ELN;CXCL1;DDIT3;CXCL3;HP;SMPD3;ATF3;PLA2G2A;MMP13;PTX3
26	Reperfusion Injury	6.31618E-12	PTGES;NOS2;BDKRB1;LCN2;SELP;TLR2;TNFRSF12A;FCER1G;IL6;MMP9;CXCL2;PLN;SLPI;NPPB;CXCL5;SERPINE1;ELN;CXCL1;DDIT3;HP;ATF3;C3;PTX3;TNFRSF9
27	**Aneurysm**	**1.03018E-11**	**PTGES;NOS2;MMP3;IL6;MMP9;CXCL2;NPPB;SERPINE1;ELN;RGS2;HP;GJA5;MMP13**
28	Metabolic Syndrome X	1.23258E-11	LCN2;NR4A1;SELP;TLR2;G0S2;IL6;MMP9;SLPI;STEAP4;SERPINE1;STAR;RGS2;ATF3;PLA2G2A;NR4A3;TNFRSF9;PTX3
29	endothelial cell dysfunction	1.9551E-11	NOS2;LCN2;SELP;TLR2;MMP9;IL6;CXCL2;PLN;CX3CL1;SERPINE1;STAR;ZC3H12A;RGS2;ATF3;ABCG1;GJA5;C3;PTX3
30	Brain Ischemia	2.29797E-11	PTGES;NOS2;LCN2;BDKRB1;SELP;MMP3;TLR2;MMP9;IL6;CX3CL1;SLPI;NPPB;SERPINE1;DDIT3;ZC3H12A;HP;ATF3;C3;MT2A;PTX3
31	Pulmonary Disease, Chronic Obstructive	2.4104E-11	NOS2;LCN2;CFLAR;TLR2;MMP3;IL6;MMP9;SLPI;NPPB;CXCL5;SERPINE1;ELN;CXCL1;TFRC;HP;PTX3
32	Kidney Failure, Acute	3.08801E-11	NOS2;BDKRB1;LCN2;SELP;TLR2;TNFRSF12A;IL6;MMP9;CXCL2;CX3CL1;NPPB;CXCL1;DDIT3;C3;PTX3
33	Cardiovascular Diseases	3.84133E-11	NOS2;BDKRB1;LCN2;SELP;TLR2;FCER1G;MMP9;IL6;CX3CL1;PLN;NPPB;CXCL5;SERPINE1;ELN;STAR;CD180;RGS2;HP;ABCG1;PLA2G2A;GJA5;PTX3
34	Rupture	4.19943E-11	PTGES;NOS2;LCN2;TFPI2;MMP3;TLR2;IL6;MMP9;CX3CL1;CXCL5;SERPINE1;ELN;CXCL1;DDIT3;MMP13;PTX3
35	Encephalomyelitis	4.22309E-11	PTGES;NOS2;BDKRB1;LCN2;CFLAR;SELP;TLR2;TNFRSF12A;FCER1G;MMP9;IL6;CXCL2;CX3CL1;SLPI;TSC22D3;CXCL1;CCL7;RASD1;MT2A;TNFRSF9
36	Lung Diseases	8.14707E-11	NOS2;SELP;TLR2;TNFRSF12A;IL6;MMP9;CXCL2;SLPI;NPPB;SERPINE1;ELN;CXCL1;SMPD3;ABCG1;ATF3;PTX3
37	Chronic Disease	8.4621E-11	SERPINE1;NOS2;BDKRB1;TFRC;SELP;HP;TLR2;FCER1G;IL6;CX3CL1;TNFRSF9
38	Myocardial Ischemia	1.31928E-10	NOS2;BDKRB1;NR4A1;SELP;MMP3;TLR2;IL6;MMP9;CX3CL1;NPPB;CXCL5;SERPINE1;CXCL1;DDIT3;ZC3H12A;HP;PTX3
39	Coronary Disease	1.50465E-10	SELP;MMP3;TLR2;IL6;MMP9;CX3CL1;APOC2;NPPB;SERPINE1;TERC;HP;PLA2G2A
40	vascular endothelial damage	1.51473E-10	SERPINE1;ELN;NOS2;SELP;HP;TLR2;MMP9;IL6;CXCL2;CX3CL1;SLPI;NPPB;PTX3
41	Neuralgia	1.71143E-10	PTGES;NOS2;BDKRB1;LCN2;SELP;TLR2;MMP3;IL6;MMP9;CXCL2;CX3CL1;NPPB;SERPINE1;CXCL1;SMPD3;CCL7;C3
42	Asthma	2.03083E-10	PTGES;NOS2;BDKRB1;LCN2;SELP;TLR2;FCER1G;MMP9;IL6;CXCL2;CX3CL1;SLPI;TSC22D3;CXCL5;SERPINE1;KIFC2;ELN;RGS2;HP;C3;TNFRSF9;PTX3
43	liver toxicity	2.22248E-10	NOS2;LCN2;CYP7B1;CFLAR;SELP;MMP3;TLR2;MMP9;IL6;CXCL2;CX3CL1;NPPB;CXCL5;SERPINE1;CXCL1;DDIT3;HP;ATF3;C3;MMP13;MT2A;TNFRSF9;PTX3
44	Neoplasm Metastasis	2.50041E-10	NOS2;BDKRB1;SELP;TFPI2;MMP3;IL6;OGN;CXCL2;FANCD2;CXCL5;HPGD;ELN;CXCL1;CXCL3;RGS2;BANP;SMPD3;C1QTNF3;BTG2;MMP13;EGLN3;PTGES;LAMC2;SNAI2;LCN2;NR4A1;TLR2;TNFRSF12A;FGF9;MMP9;CX3CL1;SLPI;SERPINE1;DDIT3;HP;ATF3;PLA2G2A;Tmeff2;CCL7
45	Colitis	2.86456E-10	PTGES;NOS2;LCN2;SELP;TLR2;TNFRSF12A;MMP9;IL6;CXCL2;CX3CL1;SLPI;TSC22D3;CXCL5;CYP26B1;CXCL1;DDIT3;HP;PLA2G2A;EGLN3;MMP13;TNFRSF9
46	Death	3.83107E-10	NOS2;BDKRB1;CFLAR;MMP3;IL6;CXCL2;CXCL5;ELN;CXCL1;TERC;TFRC;ZC3H12A;CD180;SMPD3;TGM1;C3;EGLN3;TNFRSF9;LCN2;NR4A1;PCP4;TLR2;TNFRSF12A;FGF9;MMP9;CX3CL1;PLN;NPPB;TSC22D3;SERPINE1;DDIT3;MAPK8IP3;STAR;HP;ATF3;PLA2G2A;GJA5;PTX3;MT2A
47	Pre-Eclampsia	4.08345E-10	NOS2;LCN2;NR4A1;SELP;TFPI2;MMP3;IL6;MMP9;NPPB;SERPINE1;CXCL1;TFRC;RGS2;HP;PTX3

Table S2-2. Enrichment analysis of the DEGs focusing on "Diseases"

	Gene Set Seed	p-value	Overlapping Entities
48	Pathologic Processes	4.2035E-10	PTGES;NOS2;LCN2;BDKRB1;TFPI2;TLR2;MMP3;FGF9;MMP9;IL6;CXCL2;CXCL5;SERPINE1;ELN;CXCL1;ZC3H12A;HP;PLA2G2A;MMP13;PTX3
49	Heart Diseases	4.31829E-10	NOS2;SPRY1;BDKRB1;CFLAR;SELP;MMP3;TLR2;TNFRSF12A;MMP9;IL6;PLN;NPPB;SERPINE1;ELN;DDIT3;ZC3H12A;ATF3;EGLN3;MMP13;PTX3
50	Inflammatory Bowel Diseases	4.46591E-10	NOS2;LCN2;BDKRB1;SELP;TLR2;MMP9;IL6;CX3CL1;TSC22D3;CXCL5;SERPINE1;CYP26B1;CXCL1;DDIT3;PLA2G2A;EGLN3;TNFRSF9
51	Subarachnoid Hemorrhage	4.7289E-10	NOS2;LCN2;DDIT3;NR4A1;SELP;HP;IL6;MMP9;NPPB;PTX3;TNFRSF9
52	Acute-Phase Reaction	4.96466E-10	PTGES;SERPINE1;CXCL1;LCN2;DDIT3;TFRC;HP;ABCG1;IL6;C3;CXCL5;PTX3
53	neuron toxicity	7.82763E-10	PTGES;NOS2;LCN2;BDKRB1;PCP4;MMP3;TLR2;MMP9;IL6;CX3CL1;NPPB;TSC22D3;SERPINE1;CXCL1;DDIT3;RGS2;SMPD3;ATF3;PLA2G2A;RASD1;C3;MT2A
54	Arthritis, Experimental	9.24168E-10	PTGES;NOS2;NR4A1;SELP;MMP3;TLR2;FCER1G;IL6;CX3CL1;TSC22D3;CXCL5;SERPINE1;CD180;C1QTNF3;MMP13;TNFRSF9
55	Vascular Diseases	9.29889E-10	NOS2;SELP;TLR2;IL6;MMP9;CX3CL1;TSC22D3;SERPINE1;ELN;CXCL1;ZC3H12A;HP;ATF3;CCL7;PLA2G2A;NR4A3;PTX3
56	Glomerulonephritis	9.32251E-10	NOS2;LCN2;SELP;TLR2;TNFRSF12A;FCER1G;IL6;MMP9;CXCL2;CX3CL1;NPPB;SERPINE1;HP;PLA2G2A
57	kidney ischemia	9.67845E-10	NOS2;CXCL1;LCN2;BDKRB1;SELP;TLR2;ATF3;TNFRSF12A;IL6;CXCL2;CX3CL1;TNFRSF9
58	Wounds	1.11253E-09	LAMC2;PTGES;NOS2;LCN2;SNAI2;SELP;MMP3;TLR2;TNFRSF12A;FGF9;MMP9;IL6;CXCL2;CX3CL1;SLPI;NPPB;SERPINE1;ELN;CXCL1;MMP13;MT2A
59	Cerebral Hemorrhage	1.1204E-09	PTGES;NOS2;SLC5A3;LCN2;TFRC;HP;TLR2;MMP3;IL6;MMP9;CXCL2
60	Peritonitis	1.1204E-09	NOS2;CXCL1;LCN2;NR4A1;SELP;TLR2;ATF3;IL6;MMP9;CXCL2;CXCL5
61	Ventilator-Induced Lung Injury	1.1322E-09	NOS2;CXCL1;CXCL3;TLR2;ATF3;IL6;MMP9;CXCL2;PTX3
62	Diabetes Mellitus	1.14417E-09	SPRY1;NOS2;BDKRB1;SELP;MMP3;IL6;CXCL5;C1QTNF3;C3;EGLN3;MMP13;TNFRSF9;SNAI2;LCN2;NR4A1;TLR2;MMP9;PLN;CX3CL1;NPPB;SERPINE1;DDIT3;HP;ATF3;ABCG1;GJA5;NR4A3;MT2A
63	neutrophil accumulation	1.53277E-09	NOS2;CXCL1;BDKRB1;CXCL3;SELP;CCL7;MMP9;IL6;CXCL2;SLPI;NPPB;CXCL5
64	Brain Injuries	1.71647E-09	PTGES;NOS2;BDKRB1;NR4A1;SELP;MMP3;TLR2;TNFRSF12A;MMP9;IL6;CXCL2;CX3CL1;NPPB;SERPINE1;CXCL1;DDIT3;HP;C3;MT2A
65	Lung Neoplasms	2.50908E-09	NOS2;CFLAR;SELP;TFPI2;MMP3;IL6;CXCL2;PLCL1;CXCL5;HPGD;CXCL1;TFRC;TGM1;MMP13;PTGES;SNAI2;LCN2;NR4A1;TLR2;FGF9;MMP9;CX3CL1;SLPI;SERPINE1;ATF3;PLA2G2A;Tmeff2;PTX3
66	Liver Neoplasms	2.65318E-09	PTGES;NOS2;LCN2;NR4A1;SELP;MT1A;TFPI2;TLR2;TNFRSF12A;MMP9;IL6;CX3CL1;CXCL5;SERPINE1;CXCL1;DDIT3;TFRC;TERC;ATF3;CCL7;Tmeff2;NR4A3;MMP13
67	Heart Failure	2.6563E-09	NOS2;LCN2;BDKRB1;SELP;TLR2;MMP3;TNFRSF12A;FGF9;G0S2;MMP9;IL6;PLN;CX3CL1;NPPB;SERPINE1;ELN;DDIT3;RGS2;ATF3;MMP13;PTX3
68	Shock, Septic	2.70088E-09	NOS2;BDKRB1;SELP;TLR2;IL6;MMP9;CX3CL1;SLPI;ZC3H12A;ATF3;PLA2G2A;C3;PTX3
69	Infection	2.7767E-09	NOS2;CFLAR;SELP;MMP3;G0S2;IL6;CXCL2;CXCL5;ELN;CXCL1;TFRC;BTG2;C3;TNFRSF9;PTGES;LCN2;PCP4;TLR2;TNFRSF12A;FCER1G;MMP9;CX3CL1;SLPI;NPPB;TSC22D3;SERPINE1;SH3KBP1;Fv1;HP;ATF3;PLA2G2A;CCL7;PTX3;MT2A
70	Idiopathic Pulmonary Fibrosis	2.8049E-09	SERPINE1;NOS2;LCN2;BDKRB1;TERC;MMP3;IL6;MMP9;CXCL2;BTG2;CXCL5
71	neuroinflammation	3.5691E-09	PTGES;NOS2;LCN2;BDKRB1;MMP3;TLR2;IL6;MMP9;CX3CL1;TSC22D3;SERPINE1;CXCL1;SMPD3;PLA2G2A
72	Aortic Aneurysm, Abdominal	4.05203E-09	PTGES;SERPINE1;NOS2;ELN;HP;MMP3;TLR2;TNFRSF12A;IL6;MMP9;MMP13
73	Premature Birth	4.10295E-09	SERPINE1;HPGD;NOS2;TLR2;MMP3;IL6;MMP9;SLPI;NPPB;CXCL5
74	Hypersensitivity	4.34482E-09	PTGES;NOS2;SPRY1;BDKRB1;LCN2;NR4A1;SELP;MMP3;TLR2;FCER1G;MMP9;IL6;PLCL1;CX3CL1;FANCD2;CXCL5;SERPINE1;CXCL1;TFRC;HP;C3;MT2A
75	Periodontal Diseases	4.46301E-09	NOS2;TLR2;MMP3;IL6;MMP9;SLPI;MMP13;CXCL5;PTX3
76	Thrombosis	4.89533E-09	NOS2;LCN2;SELP;TFPI2;MMP3;TLR2;FCER1G;IL6;MMP9;NPPB;SERPINE1;RNASE1;ELN;C3;MMP13;PTX3
77	Spinal Cord Injuries	5.05249E-09	PTGES;NOS2;LCN2;SELP;TLR2;IL6;MMP9;CX3CL1;SLPI;TSC22D3;ELN;CXCL1;DDIT3;PLA2G2A;MMP13
78	Pneumonia	5.74906E-09	NOS2;BDKRB1;LCN2;SELP;TLR2;IL6;MMP9;CXCL2;SLPI;NPPB;CXCL5;SERPINE1;ELN;CXCL1;DDIT3;ABCG1;ATF3;CCL7
79	Graft Rejection	7.32828E-09	NOS2;CFLAR;SELP;TLR2;IL6;MMP9;CXCL2;CX3CL1;NPPB;CXCL5;SERPINE1;HP;ATF3;C3;TNFRSF9
80	Ventricular Dysfunction	7.53752E-09	NOS2;ZC3H12A;MMP3;TNFRSF12A;IL6;MMP9;PLN;NPPB
81	Fibrosis, Liver	8.22549E-09	NOS2;MMP3;TNFRSF12A;TLR2;IL6;MMP9;CX3CL1;NPPB;SERPINE1;DDIT3;TFRC;HP;CCL7;C3;MMP13;MT2A
82	Fever	8.75313E-09	PTGES;NOS2;BDKRB1;LCN2;CFLAR;TLR2;IL6;MMP9;CXCL2;NPPB;TSC22D3;CXCL1;HP
83	Periodontitis	8.9099E-09	PTGES;SERPINE1;NOS2;TLR2;MMP3;IL6;MMP9;MMP13;PTX3
84	Multiple Sclerosis	8.99394E-09	PTGES;NOS2;LCN2;BDKRB1;SELP;MMP3;IL6;MMP9;CX3CL1;TSC22D3;SERPINE1;CXCL1;NFKBIZ;RGS2;CCL7;MT2A
85	Melanoma	9.55833E-09	NOS2;SPRY1;BDKRB1;SNAI2;SELP;TFPI2;TLR2;MMP3;TNFRSF12A;MMP9;IL6;CXCL2;CX3CL1;SERPINE1;RNASE1;ELN;CXCL1;CXCL3;BANP;C3;MMP13;TNFRSF9
86	Hypertension, Pulmonary	9.84904E-09	NOS2;LCN2;SELP;IL6;MMP9;CX3CL1;SLPI;NPPB;SERPINE1;HPGD;ELN;GJA5;BTG2;C3;MMP13
87	intimal hyperplasia	1.14277E-08	PTGES;NOS2;NR4A1;SELP;TFPI2;TLR2;MMP3;IL6;MMP9;CX3CL1;SERPINE1;ELN;DDIT3;CCL7
88	Keratitis	1.23278E-08	CXCL1;SELP;TLR2;IL6;MMP9;CXCL2;MMP13;CXCL5;TNFRSF9
89	Carcinoma, Squamous Cell	1.32997E-08	PTGES;NOS2;LCN2;SNAI2;TFPI2;MMP3;MMP9;IL6;CXCL2;SLPI;SERPINE1;CXCL1;TFRC;TERC;SH3KBP1;CCDC37;HP;ATF3;Tmeff2;PLA2G2A;ADH7;TGM1;MMP13
90	Liver Cirrhosis	1.33918E-08	NOS2;MMP3;TLR2;IL6;MMP9;NPPB;ELN;TFRC;TERC;HP;C3;MMP13
91	Metabolic Diseases	1.47387E-08	NOS2;SPRY1;LCN2;NR4A1;SELP;TNFRSF12A;IL6;CX3CL1;SLPI;NPPB;STEAP4;CXCL5;SERPINE1;CD180;BTG2;TNFRSF9
92	Leukocytosis	1.57398E-08	DDIT3;SELP;ABCG1;TLR2;IL6;MMP9;CXCL2;CXCL5
93	Renal Insufficiency, Chronic	1.63476E-08	SERPINE1;ELN;LCN2;RGS2;MMP3;TLR2;CCL7;IL6;MMP9;NPPB;PTX3
94	Renal Insufficiency	1.67969E-08	PTGES;NOS2;LCN2;MMP3;TLR2;IL6;MMP9;CXCL2;CX3CL1;NPPB;SERPINE1;CXCL1;STAR;HP
95	Ventricular Dysfunction, Left	1.81759E-08	NOS2;SELP;TLR2;IL6;MMP9;PLN;NPPB;EGLN3;PTX3
96	Fatty Liver	1.93304E-08	NOS2;SPRY1;LCN2;NR4A1;CYP7B1;TLR2;G0S2;IL6;MMP9;SERPINE1;CXCL1;DDIT3;HP;CCL7;C1QTNF3;C3;EGLN3;TNFRSF9
97	neutrophil depletion	2.06826E-08	NOS2;LCN2;SELP;TLR2;IL6;CXCL2;CXCL5
98	Obstetric Labor, Premature	2.32404E-08	PTGES;HPGD;NOS2;TLR2;IL6;MMP9;NPPB;PTX3
99	Brain Edema	2.33355E-08	PTGES;SERPINE1;NOS2;BDKRB1;ZC3H12A;SELP;TLR2;MMP3;IL6;MMP9;NPPB
100	cartilage degeneration	2.71077E-08	PTGES;NOS2;LCN2;DDIT3;CFLAR;MMP3;IL6;PRG4;MMP9;MMP13
